# Prediction of the Number of Activated Genes in Multiple Independent Cd^+2^- and As^+3^-Induced Malignant Transformations of Human Urothelial Cells (UROtsa)

**DOI:** 10.1371/journal.pone.0085614

**Published:** 2014-01-22

**Authors:** Scott H. Garrett, Seema Somji, Donald A. Sens, Ke K. Zhang

**Affiliations:** Department of Pathology, School of Medicine and Health Sciences, University of North Dakota, Grand Forks, North Dakota, United States of America; University of Kentucky, United States of America

## Abstract

**Background:**

Many toxic environmental agents such as cadmium and arsenic are found to be epidemiologically linked to increasing rates of cancers. In vitro studies show that these toxic agents induced malignant transformation in human cells. It is not clear whether such malignant transformation induced by a single toxic agent is driven by a common set of genes. Although the advancement of high-throughput technology has facilitated the profiling of global gene expression, it remains a question whether the sample size is sufficient to identify this common driver gene set.

**Results:**

We have developed a statistical method, SOFLR, to predict the number of common activated genes using a limited number of microarray samples. We conducted two case studies, cadmium and arsenic transformed human urothelial cells. Our method is able to precisely predict the number of stably induced and repressed genes and the number of samples to identify the common activated genes. The number of independent transformed isolates required for identifying the common activated genes is also estimated. The simulation study further validated our method and identified the important parameters in this analysis.

**Conclusions:**

Our method predicts the degree of similarity and diversity during cell malignant transformation by a single toxic agent. The results of our case studies imply the existence of common driver and passenger gene sets in toxin-induced transformation. Using a pilot study with small sample size, this method also helps microarray experimental design by determining the number of samples required to identify the common activated gene set.

## Introduction

Transcriptome studies and the sequencing of gene coding regions obtained from human tumors have revealed that the gene expression show both similarity and diversity for a given tumor type. Tumors were found to be highly heterogeneous with distinct gene expression patterns when compared both individual tumors [1], [2], [3] or within a single cancer [4], [5]. Nevertheless, for a specific tumor type, the gene expression often showed similarity between individual tumors in that consensus mutations [6], [7], [8] or recurrent genetic changes [9], [10], [11] were observed. One question that is unknown regarding tumor homogeneity is the role that exposure to a single environmental agent might have on the gene expression profile during the process of malignant transformation. There are few examples in the literature that illustrate the degree of similarities or alterations in gene expression that might be expected when a single toxicant is used to effect multiple independent transformations of the same cell line.

One popular paradigm of cancer development and progression is based on the concept of “driver” and “passenger” mutations [12], [13]. Driver mutations are those that confer a selective growth advantage to the cell while “passenger mutations” are those that do not confer a selective advantage but occurred as a result of acquired genomic instability. Passenger mutations are much more common than driver mutations and a major difficulty arises in separating the few driver mutations from the large background of passenger mutations. If common driver mutations exist in transformed cells, a constant number of induced and repressed genes, as the product of pathways affected by driver mutations, would be found over a large number of independent isolates. A large number of genomic studies have found common gene sets that were activated during toxin-induced in vivo or in vitro cell transformation [14], [15], [16], [17], [18], [19]. However, it is a question whether the common gene sets are stabilized because it is usually difficult to obtain sufficient number of independent transformations in genomic studies. In this study, we constructed a statistical model to predict the number of common induced and repressed genes based on a limited number of independent isolates. In order to validate our model, we did two case studies for the malignant transformation of the human urothelial cell line, UROtsa.

We are using UROtsa cell line, which can be transformed by the environmental agents, cadmium and arsenic, as the model system. The UROtsa cell line is derived from a primary culture of human urothelial cells that was immortalized using the SV40 large T-antigen [20]. The UROtsa cells grow as a contact inhibited monolayer and are not tumorigenic as judged by the inability to form colonies in soft agar and tumors in nude mice. This laboratory showed that UROtsa cells grown in a serum-free growth medium displayed features consistent with the intermediate layer of the urothelium [21]. This laboratory has also shown that the UROtsa cells can be directly malignantly transformed by exposure to Cd^+2^ or As^+3^ and that the tumor transplants produced by the transformed cells displayed histologic features consistent with human urothelial cancer [22]. Subsequently, the laboratory has isolated 5 additional As^+3^ and 6 additional Cd^+2^ transformed UROtsa cell lines and shown each to have cell culture characteristics similar to those of the original isolates, as well as to produce tumors with a histology similar to that of the original isolates [23], [24]. These additional cell lines were isolated simultaneously from independent flasks of parental UROtsa cells and were exposed to identical cell culture reagents and stock solutions of Cd^+2^ and As^+3^.

The goal of the current study was to assess consistency and stochasticity in global gene expression signatures from repeated transformation from a single carcinogen i.e. Cd^+2^ or As^+3^. Using the global gene expression data, a stochastic model for the convergence of a common activated gene set as the number of transformed cell isolates increased was constructed.

## Results

### Transformed isolates have carcinogen-specific gene expression patterns

**Figure 1:**
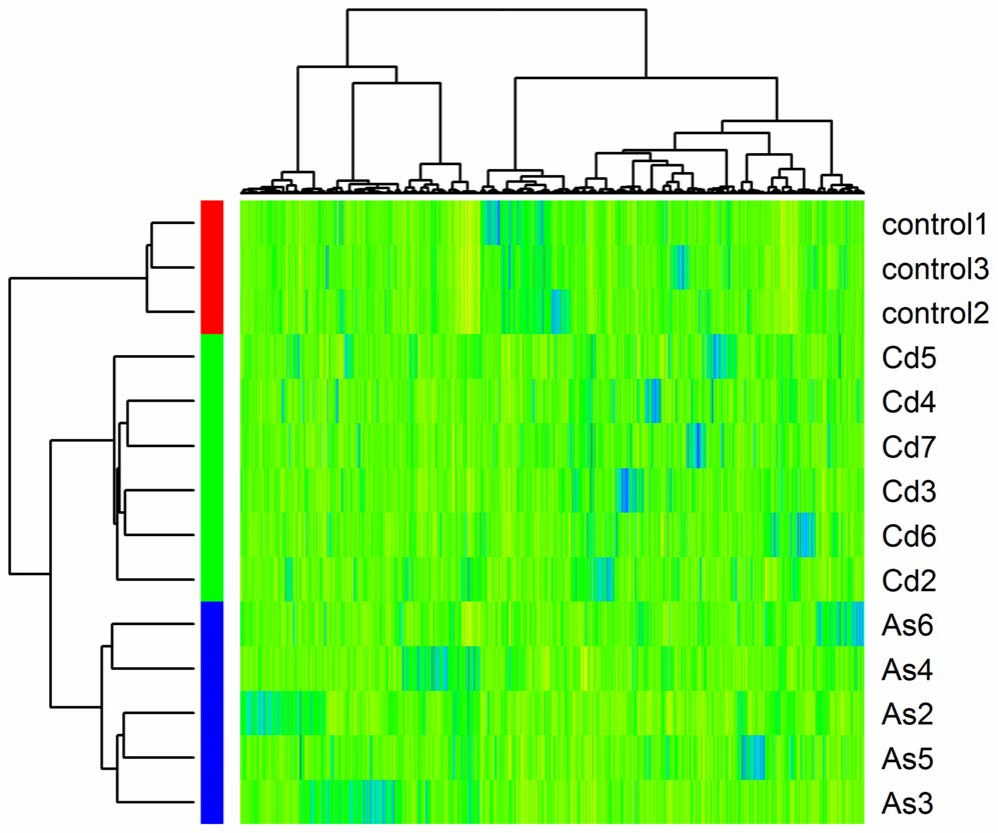
Heat map. The heat map of all UROtsa As^+3^ and Cd^+2^ transformed isolates with the non-transformed parental controls in comparison to that of As^+3^ transformed UROtsa cells. The map consists of 4,454 probes that exhibited the greatest variation in expression across isolates. The non-transformed controls and transformed isolates formed three distinct groups by hierarchical clustering.

Global gene expression analysis was performed on the 6 Cd^+2^ transformed, 5 As^3+^ transformed cell lines and 3 non-transformed parental control cell lines using the Affymetrix 133 Plus 2.0 chip. The MAS 5.0 algorithm reported that 25,074 probes were present in at least one isolate. In order to determine whether the three groups of cell lines have distinct gene expression patterns, we selected 4,454 probes that had a standard deviation greater than 0.5 across all isolates. These 4,454 probes were used for hierarchical cluster analysis using Pearson's dissimilarity and the Ward link method. In Figure 1, a heat map demonstrated that the non-transformed UROtsa control cell lines, the Cd^+2^-transformed and the As^3+^ cell lines formed three distinct groups by hierarchical clustering; suggesting higher relatedness in gene expression profiles within each group than between each group.

Empirical Bayes model was used for the differentially expressed genes by comparing transformed cell lines with parental controls. By controlling a false discovery rate (FDR) of 5%, 609 genes were found to be induced (Table S1) and 579 repressed in Cd^+2^ transformed cells (Table S2). The DEGs in As^+3^ transformed cells are 228 (Table S3) and 105 for induced and repressed (Table S4), respectively. These results indicate the transformed cell lines form specific gene expression patterns that distinguish them from parental cells and from cells transformed by a different toxic agent.

### Common genes are activated across independently transformed cell lines

**Figure 2:**
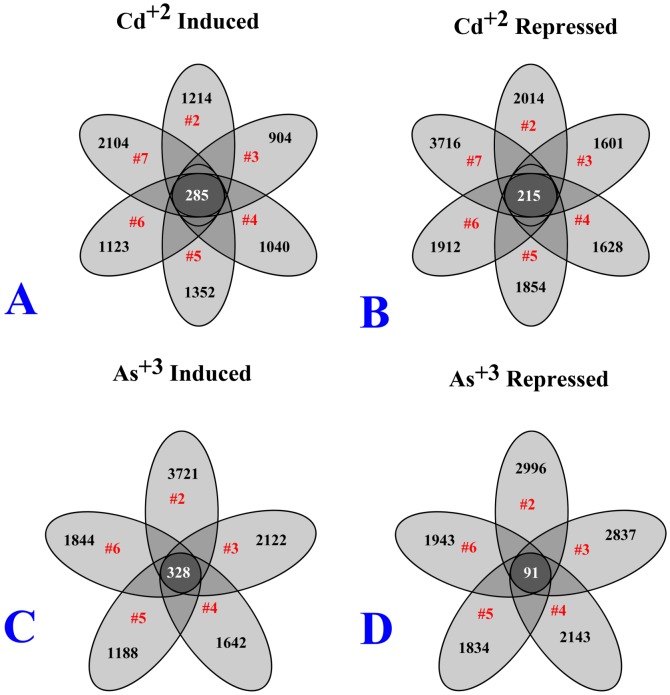
Venn diagram for the number of gene expression changes in metal transformed UROtsa cells. The Venn diagram depicts the number of Affymetrix 133 Plus 2.0 array probes that exhibit a two-fold change in gene expression in each metal-transformed UROtsa isolate compared to non-transformed UROtsa cells. The transformed isolate number is also depicted within each oval in red, and the probe overlap (number of probes expressed in all isolates) is shown in the middle of each rosette.

The number of induced and repressed array probes, defined as a two-fold induction or repression compared to the control, were collated on a gene-by-gene basis for each cell line. The number of array probes induced in the Cd^+2^ transformed cell lines compared to the non-transformed control ranged from 1,189 to 2,389 with 285 probes having common induction among all the Cd^+2^ transformed cell lines (Fig. 2A). The number of array probes repressed in the Cd^+2^ transformed cell lines compared to control lines ranged from 1,816 to 3,931 with 215 probes having common repression among all the Cd^+2^ transformed cell lines (Fig. 2B).

The number of array probes induced in the As^+3^ transformed cell lines compared to the non-transformed control ranged from 1,516 to 4,049 among the 5 cell lines with 328 probes induced in common among all 5 As^+3^ transformed cell lines (Fig. 2C). Similarly, the number of repressed probes ranged from 1,925 to 3,087 for all the cell lines with 91 probes having common repression among all the As^+3^ transformed cell lines (Fig. 2D).

It is a question whether the common genes shared by all transformed isolates are formed by random chance? We conducted a simulation study to answer this question. For 285 induced probes in the 6 Cd^+2^ transformed cells, we randomly generated 6 probe subsets from a total of 25,074 probes. The numbers of probes in the 6 subsets were the same to the numbers of induced probes of the 6 Cd^+2^ isolates, which were 1189, 1325, 1408, 1499, 1637, 2389, respectively. The overlapping probes that were shared in the 6 subsets were then identified and counted. The simulation was repeated 1,000,000 times, and none of the numbers of overlapping probes was greater than 285. So we concluded that the number of common induced probes, 285, was significantly higher than what was expected from random draws, and the p-value was less than 1E-6. The simulation studies were performed for all the 4 scenarios, induced and repressed for Cd^+2^ or As^+3^ transformation, and we found that the observed numbers of overlapping probes were all significant with p-values less than 1e-6.

### Array validation

Many of the genes identified as differentially expressed in all 6 of the Cd^+2^ transformed cell lines compared to the UROtsa parent were also independently validated for their expression. In this analysis, 338 differentially expressed genes were validated in triplicate using real time PCR. There were several criteria used for selection of the gene list for validation. Since the Affymetrix 133 Plus 2.0 chip does contain multiple probes for some individual genes, this redundancy was removed with only one gene chosen for validation. In general, any gene having a 3.2 fold alteration in expression compared to control was chosen for analysis, although there were several genes where difficulties were found in probe design or where informatics information was suspect. The numbers of genes chosen for validation are shown as the first row of Table 1. The validation was performed by testing whether the confidence interval (CI) of fold changes covered 0 or not. Overall, only 8 genes out of 161 (4.97%) in the cadmium transformed cells and 22 out of 334 in the arsenic transformed cells failed to validate. The results are near the 5% discovery rate used in the DEG list.

**Table 1:** Microarray validation using a customized qPCR array.

	Cadmium	Arsenic
n.genes evaluated	161	334
n.genes not validated	8	22
Percent not validated	4.97%	6.59%

### Statistical prediction of the number of common altered genes

**Figure 3:**
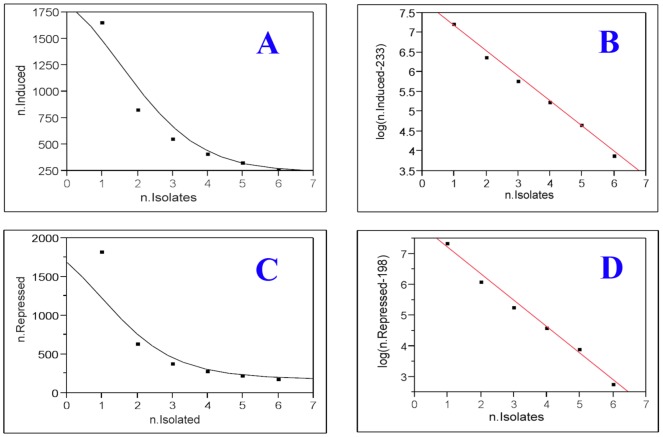
Model fitting of the number of overlapping probes for Cd^+2^ transformed cells. The x axis is the number of isolates, n.isolates, and the y-axis is the number of overlapping probes in original scale or in logarithm scale. **A** and **C**: 4P logistic curve fitting for the overlapping probes. **B** and **D**: the optimal linear fitting for the overlapping probes at logarithm scale.

The above analysis suggests that there may be a set of genes that are induced and repressed in common in Cd^+2^ or As^+3^ transformed UROtsa cells. The predicted size of each set of these in common genes among the cell lines can be predicted by hypothetically increasing the number of cell lines within each group. Two outcomes are possible: 1) the overlap falls to zero and there would not be any set of genes in common among a much larger set of cell lines or 2) the number of induced and repressed genes in common would approach a constant minimum and represent a core set of genes induced or repressed in common in the expanded set of transformed cell lines. In the case of the probes identified as induced in the Cd^+2^ transformed cell lines, the number of probes found to be induced decreased from a mean of 1,673 for a single cell line to 850 for probes induced in common in two cell lines, and 574, 433, 346, and 285 for three, four, five, and six cell lines, respectively. A fitted nonlinear line using a 4-parameter logistic model was used to predict the number of genes that would be induced in the Cd^+2^ transformed UROtsa cells (Fig. 3A). This analysis showed that as the number of isolates were increased, the curve would stabilize at approximately 230 genes induced in common for the Cd^+2^ transformed UROtsa cells. To verify this result, we used the method SOFLR as described in Methods section to fit the data in a logarithm scale using the following model,
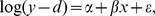
where *y* is the number of induced genes for *x* isolates, *d* is a constant integer, *α* and *β* are constant parameters, and *ε* is the random error that is normally distributed with mean 0 and standard deviation *σ*. It is obvious that y will converge to *d* as the number of isolates *x* increases. For *y* = 285 at *x* = 6, *d* ranges between 0 and 284, and an adjusted R^2^ for the linear regression can be calculated for each *d* value. It was calculated that *d* = 233 gave the best R^2^ value, 0.993 (Fig. 3B). Thus, SOFLR predicted that the number of genes induced in common among a large number of cadmium transformed cell lines would be 233. It followed that for 14 independent isolates, the predicted 95% confidence interval for *d* is (233, 233.359); therefore, the 233 driver genes induced in common could be identified using 14 independent Cd^+2^ transformed cell lines. Using this SOFLR method, we predicted that 198 genes are repressed in common for the Cd^+2^ transformed cell lines (Fig. 3C–D).

**Figure 4:**
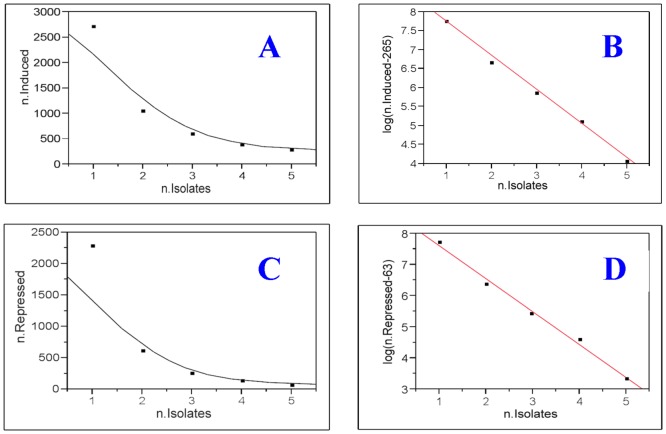
Model fitting of the number of overlapping probes for As^+2^ transformed cells. The x axis is the number of isolates, n.isolates, and the y-axis is the number of overlapping probes in original scale or in logarithm scale. **A** and **C**: 4P logistic curve fitting for the overlapping probes. **B** and **D**: the optimal linear fitting for the overlapping probes at logarithm scale.

The same analysis was performed for As^+3^ transformed cell lines. For *y* = 328 at *x* = 5, *d* ranges between 0 and 327, and we calculated the adjusted R^2^ for each regression line for a given *d* value. It showed that *d* = 265 gave the best R^2^ value that was equal to 0.993 (Fig. 4B). Thus, the statistical model, SOFLR, predicted that the number of genes induced in common among a large number of As^+3^ transformed cell lines would be 265. For 11 cell isolates, the predicted 95% confidence interval was (265, 265.459); therefore, the 265 driver genes that were consistently induced could be identified using at least 11 independent As^+3^ transformed cell lines. Using the same approach, we predicted that 63 genes were repressed in common for the As^+3^ transformed cell lines and that 9 isolates would be sufficient to identify the repressed genes in common (Fig. 4C–D).

### Simulation study

**Figure 5:**
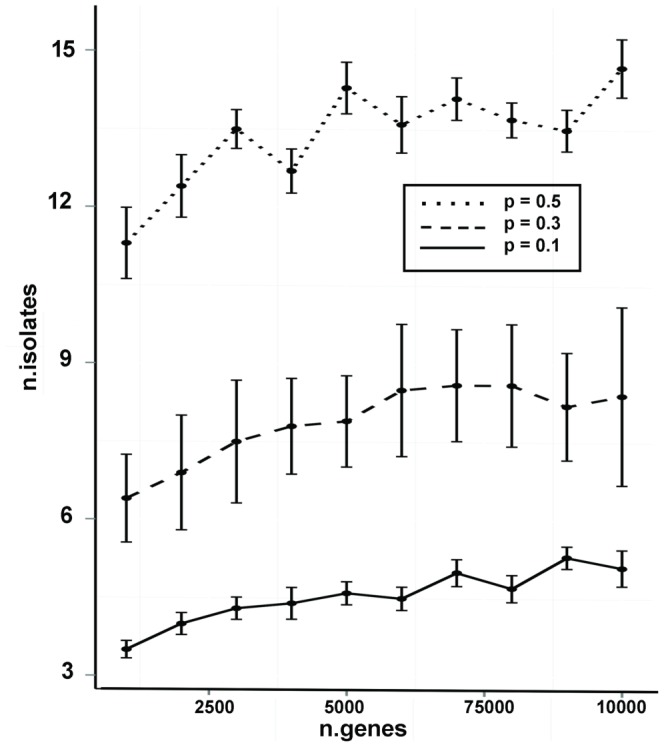
Simulation analysis for the minimum number of isolates. The x axis is the total number of genes in the passenger set, and the y axis is the number of isolates required for identifying a stable set of altered genes. The error bars show the standard errors for 10 replicates.

How many independent isolates are required for obtaining a stable set of altered genes or the driver gene set as described above? This number depends on how fast the number of overlapping genes that are changed in all isolates converges to a constant number as the number of isolates increases. Our analyses of the two case studies above showed that a relative small sample size was sufficient. Simulation was used to verify this prediction. Defining the total number of genes in the passenger set as T and the probability that a passenger gene altered in an isolate is p, the number of induced or repressed passenger genes have a mean of Tp for a given isolate. Based on a binomial distribution, n samples of passenger genes were simulated for predetermined T and p. We want to find the minimum number of n such that the number of overlapping passenger genes becomes zero. The simulation result is shown in Figure 5. The x axis is T, and the y axis is the minimum number of n. We tested three different p, 0.1, 0.3, and 0.5. Each combination of T and p was repeated 10 times and the error bars show the standard errors. Figure 5 suggests that the size of passenger set has a smaller effect on the number of isolates than the value of p. For example, for T = 5000, the number of isolates was 5 for p = 0.1, 8 for p = 0.3, and 14 for p = 0.5. The predicated numbers in our case studies are within the range of simulated results.

## Discussion

A possible explanation for the presence of a common set of induced and repressed genes among the Cd^+2^ or As^+3^ transformed cell lines would be based on the concept of “driver” and “passenger” mutations in cancer development and progression [12], [13]. In the present study it is very possible that the constant number of induced and repressed genes found over a large number of independent isolates is the product of pathways affected by driver mutations. This can be assumed since only driver mutations would lead to common pathway alterations between the cell lines whereas the more numerous passenger mutations would lead to random pathway activation in each isolate. The current data also indicate that many of the genes that were not common to all isolates had some degree of non-random character in their probability of being differentially expressed in the transformed isolates. This indicates that there may be different combination of genes that are required to support the transformed phenotype. This could manifest as either one or several genes within a pathway or gene family that would be required rather than an actual requirement of a specific gene.

The hypothesis of different combination of genes in the transformed cells promoted us to develop a statistical model for identifying the number of genes activated by the driver mutations. The high cost of high-throughput technology and the difficulty of cell transformation do not allow researchers to obtain gene expression profiles for a large number of independent transformed isolates. We aimed to predict the size of a driver gene set or the number of commonly activated genes using a small number of isolates. The fit of the linear model was near perfect (R^2^>0.99), indicating the presence of driver and passenger gene sets and the robustness of the SOFLR method. The narrow range of 95% confidence interval for the predicted number of driver genes showed the capacity of accurate prediction using SOFLR. The large number of genes and the relative small number of samples in global gene expression study presents a major challenge for statistical testing. Although the small sample size does not allow sufficient statistical power for identifying targeted genes, this study showed that the limited information can be used to accurately predict the number of consistently altered genes.

The SOFLR method can also be used for experimental design. Financial constraint remains a major factor preventing researchers to conduct genomic experiments using a large number of samples [25]. Traditional methods for determining microarray sample sizes rely on a given arbitrary effect size or an arbitrary number of targeted significant genes using some statistical criteria such as FDR [26]. Thus, the resulting sample size is likely to be under- or over-estimated depending on the given conditions. In our analysis, SOFLR is able to objectively find the minimum required number of samples for an efficient experiment design. For example, we showed that 11 As^+3^ isolates would be sufficient to identify the common induced genes and additional isolates would not help to improve the result. The high agreement of model fitting and the small variation in the simulation study indicated that our method can accurately determine the required sample size. So SOFLR helps researchers to plan their experiments more efficiently and economically.

Cadmium and arsenic are two important environmental toxic agents that can induce multiple disease effects including cancers. Cadmium has been classified as a human carcinogen by the International Agency for Research on Cancer and administration of the metal to animals results in tumors of multiple organs and tissues [27]. The initial classification of cadmium as a cancer causing agent in humans was based on an elevated incidence of lung cancer in occupational groups with evidence of elevated exposure to cadmium through inhalation. While data on the role of environmental cadmium exposure with the development of specific human cancers is limited, the study by Kellen and coworkers [28], [29] does provide epidemiological support for a role for cadmium in the development of bladder cancer. Arsenic is a metalloid with a ubiquitous distribution within the environment. Chronic exposures to low concentrations of arsenic have been associated with increased risk for the development of skin, lung and bladder cancer [29], [30], [31]. The biological processes underlying the ability of inorganic arsenic to transform human cells is unknown, but a wide-range of processes have been implicated; including oxidative stress, increased cell proliferation, inhibited DNA repair, genotoxicity, and altered cellular signaling [32], [33].

The potential of using global gene analysis on multiple independent cell lines exposed to a specific environmental agent to narrow the search for biomarker identification is reinforced by the findings in the present study using Cd^+2^ and As^+3^. The gene expression profiles were validated by our customized qPCR array, suggesting the high quality and repeatability of our microarray experiments. Overall, the balance between diversity and similarity should provide a rich environment for future biomarker discovery.

## Methods

### Cell Culture

Stock cultures of the parental UROtsa cell line were maintained in 75 cm^2^ tissue culture flasks using Dulbecco's modified Eagle's meium (DMEM) containing 5% v/v fetal calf serum in a 37°C, 5% CO_2_: 95% air atmosphere [21]. The UROtsa cell line was initially derived by immortalization of human urothelial cells via SV40 large T antigen by a previous lab [21]. The isolation and growth of the 6 additional isolates of the Cd^+2^ transformed UROtsa cells and 5 additional isolates of the As^+3^ transformed UROtsa cells have been described previously [20], [22], [23]. The cells were grown and maintained using identical conditions. Confluents flaks at a 1∶4 ratio using typsin-EDTA (0.05%, 0.02%) and the cells were fed fresh growth medium every 3 days.

### Microarray

Global gene expression analysis was performed by Genome Explorations Inc. (Memphis, TN). For cRNA synthesis and labeling, the RNA was processed and labeled according to standard RTIVT methods as described previously [34]. The fragmented cRNA was hybridized for 16 h at 45°C to GeneChip Human Genome U133 Plus 2.0 arrays (Affymetrix, Santa Clara, CA). The arrays were stained with phycoerythrein-conjugated streptavidin (Invitrogen, Carlsbad, CA) and the fluorescence intensities were determined using a GCS 3000 7G high-resolution confocal laser scanner (Affymetrix). The scanned images were analyzed using programs resident in GeneChip Operating System v1.4 (GCOS; Affymetrix). Quality control metrics for cRNA integrity, sample loading, and variations in staining were determined after background correction and signal summarization by MAS 5.0 statistical algorithms resident in GCOS and standardization of each array by global scaling the average of the fluorescent intensities of all genes on an array to a constant target intensity (TGT) of 250.

### Array validation

Of the 285 genes that were induced and 215 genes that were repressed in all 5 As^+3^ transformed cell lines compared to control UROtsa cells, those genes that were greater than 3.2 fold induced or repressed were advanced to validation by real-time PCR using a custom 384-well PCR array developed by SABiosciences (now a subsidiary of Qiagen, Valencia CA). Most of the primers were custom designed to hybridize as close as possible to the target sequence of the Affymetrix 133 Plus 2.0 array probe to avoid alternative splicing differences in the mRNA. Each primer set was empirically validated for specificity and amplification efficiency. Each sample was analyzed from RNA purified from triplicate cultures of each As^+3^ transformed isolate and parental UROtsa cells. Data analysis was performed using web-based software provided by the manufacturer (http://pcrdataanalysis.sabiosciences.com/pcr/arrayanalysis.php) and is based on the ΔΔC_T_ method. Gene expression was normalized to the geometric mean of the expression of the five reference genes, ACTB, B2M, HPRT1, RPLP0, and UBC. The assays were performed by the manufacturer. For genes initially discovered as two-fold induced or greater by the Affymetrix 133 array, the validation criteria consisted of assessing whether the upper limit of the 95% confidence interval (CI) of the mean fold induction of all five As^+3^ transformed isolates obtained from the PCR-array was above 2.0. For those genes initially discovered as repressed (expression below 0.5 fold), validation criteria was based on whether the lower limit of the CI was above 0.5.

### Microarray data analysis

Hierarchical clustering and principal components analysis were used to assess the similarity and variation across isolates. The fold change of each probe in each array from a transformed cell line was calculated over its average expression level in the parental UROtsa cell line. The activated probes were identified as having a fold change greater than 2. In order to test whether transformed isolates have a nonrandom set of overlapping probes, we derived a probability function for the random number of overlapping probes, and used simulation test to find the statistical significance of the observed number of overlapping probes. Differentially expressed probe sets (DEGs) were identified using empirical Bayes (EBayes) method [35] and the p-values were adjusted using false discovery rate [36]. The analyses were carried out using R programming language and SAS JMP® software.

### Statistical model

We divide the complete gene lists into three subsets, driver, passenger and non-responsive, depending on their responses to the environmental toxin after cell transformation. The driver genes in this article are defined as those that are consistently induced or repressed in all independently transformed isolates, potentially as the product of pathways affected by driver mutations. Thus the number of genes in the driver set is constant across all isolates, which is denoted as *d*. In contrast, the non-responsive genes are those genes that lacked response to metal-induced transformation. The passenger gene set include the genes that are randomly altered, presumably, but have a probability, *p*, to be induced or repressed in a transformed isolate. Therefore, the number of passenger genes observed in a given isolate follows a binomial distribution. Denoting the total number of passenger genes as *T*, the mean number of passenger genes is *T*p. It follows that the numbers of overlapping passenger genes between 2, 3, 4, …, n isolates would be *T*p*^2^*, *Tp^3^*, *Tp^4^*, *…*, *Tp^n^*, respectively, and the number of activate genes among n isolates would be *d+Tp^n^*. Because *T* is a constant and *p* is a probability value between 0 and 1, the number of overlapping passenger genes would be decreasing as the number of isolates increases. The quantity, *T*p*^n^*, would eventually drop below 1 for a sufficient large n, implying the number of overlapping passenger genes is approaching zero. Therefore, the common activated gene set becomes the driver gene set and the number of common activated genes is *d* when the number of independent transformed isolates is sufficiently large. We are able to predict *d* using a method called Sequentially Optimizing the Fitting of Linear Regression (SOFLR).

In order to predict *d* with a small number of isolates, we developed a statistical model to fit the number of overlapping activated genes. Suppose *n* isolates are independently transformed. The average numbers of activated genes that overlap for 1, 2, 3, …, n samples are *y_1_, y_2_, y_3_, …, y_n_*. Suppose *d* is known, then *y_1_−d, y_2_−d, …, y_n_−d* can be used to estimate *T*p, *Tp^2^, …, Tp^n^*. By logarithm transformation, log(*y_1_−d*), log(*y_2_−d*), …, log(*y_n_−d*), would be estimates for log(T)+log(p), log(T)+2log(p), …, log(T)+nlog(p), respectively. Because T and p are constant, log(*y_1_−d*), log(*y_2_−d*), …, log(*y_n_−d*) can be fitted using a linear regression model, log(y−d) = α+βx+ε, where x is the number of isolates, y is the number of observed overlapping genes altered in all x isolates, α and β are constant, and ε is the error term. SOFLR estimates *d* by sequentially testing all possible *d* values that are integers range from 0 to y−1. The value that gives the best fitting of the linear regression model would be selected as the estimated *d*. For the two case studies of Cd^+2^ and As^+3^ transformed microarray experiments, we used SOFLR to find the optimized *d* for the linear models of common induced or repressed genes.

## Conclusions

We have developed a statistical method, SOFLR, to predict the number of common activated genes in toxin-induced cell malignant transformation using a small number of isolates. The method was applied to two case studies, the cadmium and arsenic transformed UROtsa cells and showed a high degree of model fitting and accuracy of prediction. The results were also confirmed by the simulated analysis. This study showed that independent malignant cell transformations shared common gene expression patterns that implied common driver genes/mutations exist for a single toxin agent. Our method is able to accurately predict the number of common activated genes based on a relative small sample size. This will facilitate the experiment design for microarray study in order to identify the common activated gene set.

## Supporting Information

Table S1 **The list of genes significantly induced by Cadmium in Human Urothelial Cells.** The table shows the Affymetrix probe IDs, the gene symbols, the fold changes and the false discovery rates.
(DOCX)

Table S2 **The list of genes significantly repressed by Cadmium in Human Urothelial Cells.** The table shows the Affymetrix probe IDs, the gene symbols, the fold changes and the false discovery rates.
(DOCX)

Table S3 **The list of genes significantly induced by Arsenic in Human Urothelial Cells.** The table shows the Affymetrix probe IDs, the gene symbols, the fold changes and the false discovery rates.
(DOCX)

Table S4 **The list of genes significantly repressed by Arsenic in Human Urothelial Cells.** The table shows the Affymetrix probe IDs, the gene symbols, the fold changes and the false discovery rates.
(DOCX)
