# Supplementary material for: Prediction of the Number of Activated Genes in Multiple Independent Cd+2- and As+3-Induced Malignant Transformations of Human Urothelial Cells (UROtsa)
Source: PLoS One. 2014 Jan 22;9(1):e85614. doi: 10.1371/journal.pone.0085614 (PMC3899011; doi:10.1371/journal.pone.0085614)
Supplement: Table S1 — The list of genes significantly induced by Cadmium in Human Urothelial Cells. The table shows the Affymetrix probe IDs, the gene symbols, the fold changes and the false discovery rates. (DOCX) [file pone.0085614.s001.docx]

**Table S1**. The list of genes significantly induced by Cadmium in Human Urothelial Cells.

| **Probe** | **Gene Symbol** | **Fold Change** | **FDR** |
| --- | --- | --- | --- |
| 204540_at | EEF1A2 | 62.87 | 0.000444 |
| 224341_x_at | TLR4 | 54.50 | 0.000644 |
| 211719_x_at | FN1 | 42.43 | 0.004781 |
| 212328_at | LIMCH1 | 41.18 | 6.01E-05 |
| 203440_at | CDH2 | 34.98 | 0.000721 |
| 204470_at | CXCL1 | 32.64 | 0.013486 |
| 206953_s_at | LPHN2 | 29.81 | 0.001318 |
| 219274_at | TSPAN12 | 29.26 | 0.001918 |
| 209465_x_at | PTN | 28.90 | 0.001825 |
| 234980_at | TMEM56 | 28.79 | 1.05E-05 |
| 208358_s_at | UGT8 | 28.33 | 1.65E-05 |
| 228956_at | UGT8 | 27.43 | 0.0006 |
| 210135_s_at | SHOX2 | 23.96 | 0.000403 |
| 227449_at | EPHA4 | 23.80 | 0.008011 |
| 212464_s_at | FN1 | 22.32 | 0.002558 |
| 241703_at | RUNDC3B | 22.21 | 0.002616 |
| 227209_at | CNTN1 | 9.98 | 0.017396 |
| 222146_s_at | TCF4 | 9.95 | 0.00915 |
| 204983_s_at | GPC4 | 9.85 | 0.003175 |
| 202833_s_at | SERPINA1 | 9.72 | 0.014937 |
| 232068_s_at | TLR4 | 9.47 | 0.01086 |
| 206706_at | NTF3 | 9.42 | 0.001391 |
| 1556696_s_at | FLJ42709 | 9.11 | 0.002822 |
| 224397_s_at | TMTC1 | 9.01 | 0.00864 |
| 1560570_a_at | NA | 8.96 | 0.038298 |
| 232481_s_at | SLITRK6 | 8.94 | 0.000461 |
| 242573_at | NA | 8.84 | 0.043285 |
| 238926_at | NA | 8.74 | 0.002749 |
| 219984_s_at | HRASLS | 8.64 | 0.005236 |
| 201645_at | TNC | 8.38 | 0.001624 |
| 225681_at | CTHRC1 | 8.35 | 0.000448 |
| 233040_at | PLEKHA5 | 8.22 | 0.015118 |
| 204816_s_at | DHX34 | 7.95 | 0.000945 |
| 201719_s_at | EPB41L2 | 7.90 | 0.000798 |
| 219568_x_at | SOX18 | 7.87 | 0.005007 |
| 215193_x_at | NA | 7.86 | 0.046555 |
| 235976_at | SLITRK6 | 7.80 | 0.000893 |
| 1553924_at | FMO9P | 7.74 | 0.002025 |
| 211990_at | HLA-DPA1 | 7.72 | 0.033688 |
| 222453_at | CYBRD1 | 7.67 | 0.012235 |
| 205741_s_at | DTNA | 7.67 | 0.000327 |
| 237278_x_at | NA | 7.62 | 0.026105 |
| 212686_at | PPM1H | 7.45 | 0.000477 |
| 212325_at | LIMCH1 | 7.25 | 8.49E-05 |
| 201428_at | CLDN4 | 7.17 | 0.000352 |
| 235343_at | VASH2 | 7.10 | 0.008888 |
| 232235_at | DSEL | 7.05 | 0.000418 |
| 209466_x_at | PTN | 7.03 | 0.014789 |
| 221004_s_at | ITM2C | 6.97 | 0.004834 |
| 242069_at | CBX5 | 6.83 | 0.000989 |
| 221060_s_at | TLR4 | 6.80 | 8.04E-05 |
| 210815_s_at | CALCRL | 6.80 | 0.026678 |
| 243541_at | IL31RA | 6.78 | 0.005441 |
| 226322_at | TMTC1 | 6.50 | 0.00063 |
| 220225_at | IRX4 | 6.43 | 0.000269 |
| 1569723_a_at | SPIRE2 | 6.39 | 0.002185 |
| 211506_s_at | IL8 | 6.33 | 0.002722 |
| 227919_at | UCA1 | 6.28 | 0.006728 |
| 232176_at | SLITRK6 | 6.20 | 0.0024 |
| 221207_s_at | NBEA | 6.15 | 0.004189 |
| 225275_at | EDIL3 | 6.05 | 0.00019 |
| 209505_at | NR2F1 | 6.02 | 0.025665 |
| 201718_s_at | EPB41L2 | 5.96 | 0.00055 |
| 240856_at | NA | 5.89 | 0.001022 |
| 219566_at | PLEKHF1 | 5.83 | 0.00475 |
| 212327_at | LIMCH1 | 5.82 | 0.000628 |
| 235122_at | HIVEP3 | 5.72 | 0.002181 |
| 232760_at | TEX15 | 5.71 | 9.95E-05 |
| 225105_at | C12orf75 | 5.65 | 0.00024 |
| 228494_at | PPP1R9A | 5.64 | 0.001334 |
| 205199_at | CA9 | 5.60 | 0.020579 |
| 226751_at | CNRIP1 | 5.53 | 0.009297 |
| 211429_s_at | SERPINA1 | 5.51 | 0.008577 |
| 202986_at | ARNT2 | 5.51 | 0.011288 |
| 202668_at | EFNB2 | 5.49 | 0.001395 |
| 237515_at | TMEM56 | 5.48 | 0.015149 |
| 209312_x_at | NA | 5.46 | 0.026672 |
| 203753_at | TCF4 | 5.41 | 0.010797 |
| 227468_at | CPT1C | 5.40 | 0.010701 |
| 209619_at | CD74 | 5.28 | 0.04021 |
| 219938_s_at | PSTPIP2 | 5.18 | 0.000581 |
| 1555564_a_at | CFI | 5.18 | 0.006462 |
| 35148_at | TJP3 | 5.08 | 0.005164 |
| 219373_at | DPM3 | 5.07 | 0.001186 |
| 227654_at | FAM65C | 5.07 | 0.000835 |
| 214358_at | ACACA | 5.07 | 0.000591 |
| 207076_s_at | ASS1 | 5.06 | 2.53E-05 |
| 235371_at | GLT8D4 | 5.00 | 0.001658 |
| 219295_s_at | PCOLCE2 | 4.91 | 4.70E-05 |
| 1553605_a_at | ABCA13 | 4.90 | 0.029943 |
| 204798_at | MYB | 4.90 | 0.001028 |
| 230003_at | NA | 4.89 | 0.035961 |
| 212386_at | TCF4 | 4.87 | 0.010361 |
| 209173_at | AGR2 | 4.87 | 0.007225 |
| 225971_at | DDHD1 | 4.86 | 0.001032 |
| 205542_at | STEAP1 | 4.78 | 0.009997 |
| 208306_x_at | HLA-DRB4 | 4.74 | 0.042547 |
| 207361_at | HBP1 | 4.62 | 0.00784 |
| 227623_at | NA | 4.61 | 0.018215 |
| 227084_at | DTNA | 4.60 | 0.013009 |
| 1558440_at | LOC100128687 | 4.58 | 0.018911 |
| 209209_s_at | FERMT2 | 4.48 | 0.001669 |
| 235759_at | NA | 4.46 | 0.033133 |
| 216218_s_at | PLCL2 | 4.46 | 0.016808 |
| 205609_at | ANGPT1 | 4.45 | 0.001988 |
| 225970_at | DDHD1 | 4.42 | 0.002639 |
| 218731_s_at | VWA1 | 4.42 | 0.001421 |
| 221912_s_at | CCDC28B | 4.41 | 0.006105 |
| 220779_at | PADI3 | 4.36 | 0.013081 |
| 237034_at | NA | 4.32 | 0.000503 |
| 231385_at | DPPA3 | 4.32 | 0.047438 |
| 229390_at | FAM26F | 4.32 | 0.001655 |
| 203441_s_at | CDH2 | 4.30 | 0.005703 |
| 219304_s_at | PDGFD | 4.25 | 0.012386 |
| 226997_at | ADAMTS12 | 4.25 | 0.035261 |
| 220655_at | TNIP3 | 4.24 | 0.021 |
| 223821_s_at | SUSD4 | 4.23 | 0.028272 |
| 230527_at | NA | 4.21 | 0.009434 |
| 220334_at | RGS17 | 4.17 | 0.007107 |
| 218723_s_at | C13orf15 | 4.17 | 0.004935 |
| 202669_s_at | EFNB2 | 4.16 | 0.017029 |
| 214088_s_at | FUT3 | 4.15 | 0.000374 |
| 215143_at | DPY19L2P2 | 4.14 | 0.012383 |
| 211828_s_at | TNIK | 4.14 | 0.004815 |
| 205673_s_at | ASB9 | 4.13 | 0.000466 |
| 219537_x_at | DLL3 | 4.13 | 0.018826 |
| 221953_s_at | EDEM2 | 4.12 | 0.039457 |
| 242939_at | TFDP1 | 4.12 | 0.020971 |
| 238029_s_at | SLC16A14 | 4.08 | 0.004644 |
| 1555214_a_at | CLEC7A | 4.08 | 0.029856 |
| 210633_x_at | KRT10 | 4.04 | 0.000325 |
| 206826_at | PMP2 | 4.00 | 0.00782 |
| 239288_at | TNIK | 3.99 | 0.005352 |
| 207379_at | EDIL3 | 3.98 | 0.000567 |
| 214031_s_at | KRT7 | 3.94 | 0.022801 |
| 230152_at | WDR52 | 3.94 | 0.011561 |
| 219869_s_at | SLC39A8 | 3.92 | 0.009733 |
| 210261_at | KCNK2 | 3.92 | 0.033904 |
| 228087_at | CCDC126 | 3.92 | 0.042477 |
| 223204_at | C4orf18 | 3.87 | 0.034287 |
| 1553600_at | TMIE | 3.85 | 0.043662 |
| 222847_s_at | EGLN3 | 3.84 | 0.017275 |
| 213107_at | TNIK | 3.84 | 0.000758 |
| 212385_at | TCF4 | 3.83 | 0.009776 |
| 220509_at | RBM26 | 3.81 | 0.03554 |
| 203661_s_at | TMOD1 | 3.80 | 0.002765 |
| 226743_at | SLFN11 | 3.79 | 0.000428 |
| 227703_s_at | SYTL4 | 3.79 | 0.022355 |
| 229296_at | LOC100128501 | 3.76 | 0.001784 |
| 1554831_x_at | ALS2CR11 | 3.75 | 0.026006 |
| 202859_x_at | IL8 | 3.74 | 0.005093 |
| 210108_at | CACNA1D | 3.74 | 0.024511 |
| 226931_at | TMTC1 | 3.74 | 0.011411 |
| 219692_at | KREMEN2 | 3.73 | 0.010508 |
| 1562587_at | CLNK | 3.73 | 0.00541 |
| 1553138_a_at | ANKLE1 | 3.72 | 0.033064 |
| 211596_s_at | LRIG1 | 3.71 | 0.034011 |
| 228762_at | LFNG | 3.67 | 0.012639 |
| 203854_at | CFI | 3.66 | 0.017713 |
| 203700_s_at | DIO2 | 3.65 | 0.033218 |
| 219895_at | FAM70A | 3.63 | 0.027154 |
| 212820_at | DMXL2 | 3.62 | 0.000414 |
| 205803_s_at | TRPC1 | 3.62 | 0.005046 |
| 211203_s_at | CNTN1 | 3.61 | 0.036692 |
| 224797_at | ARRDC3 | 3.61 | 0.017932 |
| 219572_at | CADPS2 | 3.61 | 0.000638 |
| 209897_s_at | SLIT2 | 3.59 | 0.009067 |
| 228214_at | SOX6 | 3.59 | 0.020729 |
| 230710_at | NA | 3.59 | 0.011779 |
| 238439_at | ANKRD22 | 3.58 | 0.000642 |
| 210102_at | VWA5A | 3.55 | 0.005063 |
| 231240_at | DIO2 | 3.54 | 0.044252 |
| 205055_at | ITGAE | 3.53 | 0.000554 |
| 223642_at | ZIC2 | 3.50 | 0.000396 |
| 213943_at | TWIST1 | 3.49 | 0.014324 |
| 239302_s_at | NA | 3.49 | 0.003731 |
| 209016_s_at | KRT7 | 3.47 | 0.000244 |
| 205645_at | REPS2 | 3.47 | 0.002771 |
| 225667_s_at | FAM84A | 3.46 | 0.000128 |
| 209199_s_at | MEF2C | 3.44 | 0.030277 |
| 226069_at | PRICKLE1 | 3.43 | 0.005365 |
| 220991_s_at | RNF32 | 3.43 | 0.040687 |
| 213891_s_at | TCF4 | 3.42 | 0.018781 |
| 216488_s_at | ATP11A | 3.41 | 0.004316 |
| 1566938_at | NA | 3.40 | 0.029568 |
| 209839_at | DNM3 | 3.39 | 0.007896 |
| 206100_at | CPM | 3.38 | 0.010906 |
| 238021_s_at | CRNDE | 3.36 | 0.001356 |
| 1553708_at | MGC16075 | 3.34 | 0.005196 |
| 214028_x_at | TDRD3 | 3.32 | 0.02502 |
| 216200_at | PLEKHM1 | 3.30 | 0.022462 |
| 230446_at | NA | 3.30 | 0.005209 |
| 228651_at | VWA1 | 3.30 | 0.013639 |
| 230875_s_at | ATP11A | 3.30 | 0.008061 |
| 222483_at | EFHD2 | 3.30 | 0.003113 |
| 219683_at | FZD3 | 3.29 | 0.023128 |
| 1555756_a_at | CLEC7A | 3.28 | 0.002362 |
| 1556695_a_at | FLJ42709 | 3.28 | 0.028169 |
| 222651_s_at | TRPS1 | 3.27 | 0.001278 |
| 227088_at | PDE5A | 3.27 | 0.015211 |
| 209925_at | OCLN | 3.27 | 0.011729 |
| 224870_at | KIAA0114 | 3.26 | 0.00591 |
| 44040_at | FBXO41 | 3.26 | 0.010732 |
| 210807_s_at | SLC16A7 | 3.25 | 0.021559 |
| 243988_at | NA | 3.25 | 0.006461 |
| 227063_at | C17orf61 | 3.24 | 0.000557 |
| 210415_s_at | ODF2 | 3.24 | 0.03927 |
| 219973_at | ARSJ | 3.24 | 0.002563 |
| 240042_at | FIBCD1 | 3.23 | 0.016493 |
| 226550_at | NA | 3.22 | 0.001165 |
| 201860_s_at | PLAT | 3.22 | 0.002823 |
| 217274_x_at | MYL4 | 3.22 | 0.020156 |
| 229391_s_at | FAM26F | 3.21 | 0.005138 |
| 233946_at | SMU1 | 3.21 | 0.023011 |
| 203196_at | ABCC4 | 3.21 | 0.038345 |
| 202241_at | TRIB1 | 3.20 | 0.000425 |
| 240382_at | NA | 3.20 | 0.020553 |
| 227425_at | REPS2 | 3.20 | 0.000588 |
| 201010_s_at | TXNIP | 3.20 | 0.048259 |
| 1555950_a_at | CD55 | 3.18 | 0.037088 |
| 202478_at | TRIB2 | 3.18 | 0.026432 |
| 227840_at | C2orf76 | 3.16 | 0.001224 |
| 218317_x_at | NA | 3.15 | 0.001432 |
| 1558703_at | SLC46A1 | 3.14 | 0.008964 |
| 1560204_at | NT5DC4 | 3.14 | 0.020692 |
| 1558501_at | DNM3 | 3.14 | 0.005654 |
| 212909_at | LYPD1 | 3.14 | 0.018773 |
| 207267_s_at | DSCR6 | 3.13 | 0.005582 |
| 227867_at | LOC129293 | 3.13 | 0.002542 |
| 210176_at | TLR1 | 3.11 | 0.034721 |
| 230110_at | MCOLN2 | 3.11 | 0.020232 |
| 240084_at | CBX2 | 3.11 | 0.023226 |
| 212382_at | TCF4 | 3.10 | 0.022952 |
| 235299_at | SLC41A2 | 3.09 | 0.004289 |
| 229431_at | RFXAP | 3.09 | 0.00067 |
| 203789_s_at | SEMA3C | 3.08 | 0.00843 |
| 228551_at | DENND5B | 3.08 | 0.011083 |
| 210652_s_at | TTC39A | 3.07 | 0.025182 |
| 223798_at | SLC41A2 | 3.06 | 0.003294 |
| 213316_at | KIAA1462 | 3.05 | 0.025717 |
| 229553_at | PGM2L1 | 3.04 | 0.008897 |
| 227282_at | PCDH19 | 3.04 | 0.004204 |
| 220030_at | STYK1 | 3.03 | 0.020173 |
| 241767_at | NA | 3.03 | 0.048033 |
| 239896_at | NA | 3.03 | 0.009293 |
| 204602_at | DKK1 | 3.02 | 0.019262 |
| 217050_at | EPAG | 3.01 | 0.002918 |
| 1558643_s_at | EDIL3 | 3.01 | 0.000537 |
| 214169_at | UNC84A | 3.01 | 0.017398 |
| 210675_s_at | PTPRR | 3.00 | 0.033433 |
| 1566852_at | TRIM42 | 2.99 | 0.023174 |
| 230465_at | HS2ST1 | 2.99 | 0.03855 |
| 226278_at | SVIP | 2.99 | 0.003503 |
| 229256_at | PGM2L1 | 2.98 | 0.002843 |
| 205777_at | DUSP9 | 2.98 | 0.012351 |
| 242794_at | MAML3 | 2.98 | 0.03247 |
| 225842_at | PHLDA1 | 2.98 | 0.017447 |
| 228754_at | SLC6A6 | 2.97 | 0.010962 |
| 215813_s_at | PTGS1 | 2.97 | 0.008492 |
| 218901_at | PLSCR4 | 2.97 | 0.002955 |
| 203919_at | TCEA2 | 2.95 | 0.02979 |
| 205762_s_at | DUS4L | 2.93 | 0.032342 |
| 205180_s_at | ADAM8 | 2.93 | 0.030015 |
| 228061_at | CCDC126 | 2.92 | 0.018637 |
| 207850_at | CXCL3 | 2.92 | 0.004253 |
| 228933_at | NHS | 2.91 | 0.000621 |
| 207023_x_at | KRT10 | 2.91 | 0.000686 |
| 212094_at | PEG10 | 2.91 | 0.0052 |
| 220431_at | NA | 2.91 | 0.004213 |
| 205846_at | PTPRB | 2.89 | 0.004842 |
| 204720_s_at | DNAJC6 | 2.89 | 0.006849 |
| 212607_at | AKT3 | 2.89 | 0.005596 |
| 202149_at | NEDD9 | 2.89 | 0.013768 |
| 229704_at | NA | 2.88 | 0.004578 |
| 209210_s_at | FERMT2 | 2.88 | 0.000725 |
| 221605_s_at | PIPOX | 2.87 | 0.015265 |
| 236030_at | RCOR2 | 2.87 | 0.003542 |
| 241030_at | NA | 2.87 | 0.013554 |
| 1558308_at | FLJ33297 | 2.86 | 0.020627 |
| 228899_at | LOC100132884 | 2.85 | 0.000763 |
| 229860_x_at | C4orf48 | 2.84 | 0.00608 |
| 201811_x_at | SH3BP5 | 2.84 | 0.011475 |
| 213424_at | KIAA0895 | 2.83 | 0.01119 |
| 222735_at | TMEM38B | 2.83 | 0.000814 |
| 213287_s_at | KRT10 | 2.83 | 0.000611 |
| 212460_at | C14orf147 | 2.83 | 0.000716 |
| 238058_at | LOC150381 | 2.83 | 0.002733 |
| 209348_s_at | MAF | 2.82 | 0.042624 |
| 205802_at | TRPC1 | 2.82 | 0.003328 |
| 236225_at | GGT6 | 2.82 | 0.013511 |
| 1558322_a_at | PAQR9 | 2.81 | 0.038718 |
| 219313_at | GRAMD1C | 2.80 | 0.003225 |
| 222857_s_at | KCNMB4 | 2.80 | 0.009893 |
| 225987_at | STEAP4 | 2.80 | 0.009147 |
| 201313_at | ENO2 | 2.80 | 0.02965 |
| 244553_at | NA | 2.79 | 0.048272 |
| 213693_s_at | MUC1 | 2.79 | 0.002338 |
| 235775_at | TMTC2 | 2.78 | 0.003602 |
| 235019_at | CPM | 2.77 | 0.026634 |
| 212944_at | SLC5A3 | 2.77 | 0.0033 |
| 218502_s_at | TRPS1 | 2.75 | 0.01114 |
| 210355_at | PTHLH | 2.74 | 0.00943 |
| 227126_at | PTPRG | 2.74 | 0.003374 |
| 244535_at | NA | 2.74 | 0.004458 |
| 234081_at | NA | 2.74 | 0.004758 |
| 219562_at | RAB26 | 2.74 | 0.002351 |
| 227783_at | CCDC57 | 2.72 | 0.02943 |
| 204393_s_at | ACPP | 2.72 | 0.02449 |
| 220407_s_at | TGFB2 | 2.72 | 0.047176 |
| 228221_at | SLC44A3 | 2.72 | 0.002732 |
| 213093_at | PRKCA | 2.72 | 0.003543 |
| 242571_at | REPS2 | 2.71 | 0.002379 |
| 230250_at | PTPRB | 2.71 | 0.017788 |
| 226070_at | C9orf142 | 2.71 | 0.019132 |
| 209946_at | VEGFC | 2.71 | 0.001814 |
| 201397_at | PHGDH | 2.70 | 0.004399 |
| 239579_at | EPHX4 | 2.70 | 0.002038 |
| 229363_at | NA | 2.69 | 0.010081 |
| 227525_at | GLCCI1 | 2.69 | 0.007365 |
| 239657_x_at | FOXO6 | 2.68 | 0.009595 |
| 228400_at | SHROOM3 | 2.68 | 0.000739 |
| 221088_s_at | PPP1R9A | 2.67 | 0.007289 |
| 230369_at | GPR161 | 2.66 | 0.029365 |
| 231294_at | STT3B | 2.65 | 0.020129 |
| 206157_at | PTX3 | 2.65 | 0.043995 |
| 1557309_at | DENND1B | 2.64 | 0.04781 |
| 209504_s_at | PLEKHB1 | 2.62 | 0.028813 |
| 228381_at | ATF7IP2 | 2.62 | 0.002807 |
| 227506_at | SLC16A9 | 2.62 | 0.036213 |
| 242417_at | NA | 2.61 | 0.009554 |
| 223723_at | MFI2 | 2.61 | 0.000477 |
| 232914_s_at | SYTL2 | 2.61 | 0.005077 |
| 233527_at | NA | 2.60 | 0.033305 |
| 217478_s_at | HLA-DMA | 2.60 | 0.021753 |
| 221698_s_at | CLEC7A | 2.60 | 0.019967 |
| 59437_at | C9orf116 | 2.60 | 0.002023 |
| 235466_s_at | DISP1 | 2.59 | 0.001311 |
| 204916_at | RAMP1 | 2.58 | 0.009883 |
| 230309_at | NA | 2.58 | 0.042378 |
| 205968_at | KCNS3 | 2.58 | 0.003865 |
| 209183_s_at | C10orf10 | 2.57 | 0.004864 |
| 1558830_at | NA | 2.57 | 0.013579 |
| 205239_at | AREG | 2.56 | 0.008366 |
| 218718_at | PDGFC | 2.56 | 0.008835 |
| 235407_at | NA | 2.56 | 0.003611 |
| 222219_s_at | TLE6 | 2.56 | 0.041026 |
| 230136_at | LOC400099 | 2.56 | 0.003591 |
| 222062_at | IL27RA | 2.55 | 0.01705 |
| 230323_s_at | TMEM45B | 2.54 | 0.031595 |
| 203186_s_at | S100A4 | 2.53 | 0.001257 |
| 208237_x_at | ADAM22 | 2.53 | 0.009037 |
| 230383_x_at | NA | 2.53 | 0.029578 |
| 232383_at | TFEC | 2.53 | 0.025122 |
| 229546_at | LOC653602 | 2.53 | 0.019363 |
| 206448_at | ZNF365 | 2.53 | 0.000967 |
| 203423_at | RBP1 | 2.52 | 0.002398 |
| 231192_at | NA | 2.52 | 0.002273 |
| 238417_at | PGM2L1 | 2.52 | 0.003354 |
| 228648_at | LRG1 | 2.52 | 0.009532 |
| 243101_x_at | NA | 2.52 | 0.035862 |
| 209366_x_at | CYB5A | 2.51 | 0.003682 |
| 212315_s_at | NUP210 | 2.51 | 0.000949 |
| 1556769_a_at | NA | 2.51 | 0.016472 |
| 234331_s_at | FAM84A | 2.50 | 0.041116 |
| 1561017_at | NA | 2.50 | 0.008815 |
| 212316_at | NUP210 | 2.50 | 0.002273 |
| 226226_at | TMEM45B | 2.50 | 0.00836 |
| 50965_at | RAB26 | 2.49 | 0.001595 |
| 1555774_at | ZAR1 | 2.49 | 0.033257 |
| 228988_at | ZNF711 | 2.49 | 0.007039 |
| 203372_s_at | SOCS2 | 2.49 | 0.03848 |
| 213508_at | C14orf147 | 2.48 | 0.003098 |
| 1552519_at | ACVR1C | 2.47 | 0.008098 |
| 235490_at | TMEM107 | 2.47 | 0.039663 |
| 209909_s_at | TGFB2 | 2.47 | 0.013942 |
| 209409_at | GRB10 | 2.46 | 0.011127 |
| 213947_s_at | NUP210 | 2.46 | 0.004089 |
| 238462_at | UBASH3B | 2.46 | 0.003779 |
| 229404_at | TWIST2 | 2.46 | 0.036559 |
| 1569144_a_at | C9orf169 | 2.44 | 0.01049 |
| 232231_at | RUNX2 | 2.43 | 0.021511 |
| 209633_at | PPP2R3A | 2.43 | 0.004052 |
| 233334_x_at | NA | 2.43 | 0.009398 |
| 204042_at | WASF3 | 2.43 | 0.000924 |
| 217998_at | NA | 2.42 | 0.018693 |
| 215726_s_at | CYB5A | 2.42 | 0.001922 |
| 1569741_at | NA | 2.42 | 0.045427 |
| 237655_at | NA | 2.42 | 0.008456 |
| 204464_s_at | EDNRA | 2.41 | 0.012293 |
| 223687_s_at | LY6K | 2.41 | 0.010482 |
| 203961_at | NEBL | 2.40 | 0.007011 |
| 205128_x_at | PTGS1 | 2.40 | 0.011359 |
| 1553970_s_at | CEL | 2.40 | 0.001881 |
| 218772_x_at | TMEM38B | 2.40 | 0.00116 |
| 219580_s_at | TMC5 | 2.39 | 0.036033 |
| 212792_at | DPY19L1 | 2.39 | 0.006957 |
| 221791_s_at | CCDC72 | 2.39 | 0.001753 |
| 209607_x_at | NA | 2.39 | 0.007587 |
| 219773_at | NOX4 | 2.39 | 0.011764 |
| 244231_at | LOC149684 | 2.39 | 0.031753 |
| 228110_x_at | RABGEF1 | 2.39 | 0.025619 |
| 200907_s_at | PALLD | 2.39 | 0.006737 |
| 203562_at | FEZ1 | 2.38 | 0.026329 |
| 216383_at | NA | 2.38 | 0.037267 |
| 232899_at | NA | 2.38 | 0.004743 |
| 225670_at | FAM173B | 2.38 | 0.003897 |
| 238669_at | PTGS1 | 2.38 | 0.011341 |
| 216793_x_at | NA | 2.38 | 0.040752 |
| 210580_x_at | NA | 2.37 | 0.007692 |
| 209774_x_at | CXCL2 | 2.36 | 0.041251 |
| 205698_s_at | MAP2K6 | 2.36 | 0.009075 |
| 205190_at | PLS1 | 2.35 | 0.002303 |
| 227492_at | NA | 2.35 | 0.001398 |
| 218404_at | SNX10 | 2.35 | 0.00228 |
| 235010_at | LOC729013 | 2.35 | 0.00328 |
| 222719_s_at | PDGFC | 2.35 | 0.001885 |
| 226546_at | NA | 2.34 | 0.008245 |
| 238022_at | CRNDE | 2.34 | 0.021418 |
| 211602_s_at | TRPC1 | 2.34 | 0.021754 |
| 205092_x_at | ZBTB1 | 2.34 | 0.017078 |
| 203910_at | ARHGAP29 | 2.34 | 0.002011 |
| 228972_at | NA | 2.33 | 0.001646 |
| 223895_s_at | EPN3 | 2.33 | 0.005211 |
| 217206_at | NA | 2.33 | 0.035661 |
| 239898_x_at | ZNF286A | 2.33 | 0.008164 |
| 203723_at | ITPKB | 2.32 | 0.006545 |
| 1556181_at | hCG_20426 | 2.32 | 0.000724 |
| 1556518_at | NA | 2.31 | 0.004227 |
| 221577_x_at | GDF15 | 2.31 | 0.019767 |
| 210215_at | TFR2 | 2.31 | 0.008192 |
| 203788_s_at | SEMA3C | 2.31 | 0.041123 |
| 201328_at | ETS2 | 2.31 | 0.022451 |
| 243893_at | KCNC3 | 2.31 | 0.03487 |
| 1556182_x_at | hCG_20426 | 2.30 | 0.002418 |
| 225967_s_at | C17orf89 | 2.30 | 0.001167 |
| 224637_at | LOC100128731 | 2.30 | 0.000596 |
| 226439_s_at | NBEA | 2.30 | 0.02928 |
| 220520_s_at | NUP62CL | 2.29 | 0.004611 |
| 220201_at | RC3H2 | 2.29 | 0.0013 |
| 203028_s_at | CYBA | 2.29 | 0.00359 |
| 209105_at | NCOA1 | 2.28 | 0.00761 |
| 219263_at | RNF128 | 2.28 | 0.021406 |
| 225142_at | JHDM1D | 2.28 | 0.02293 |
| 225906_at | NA | 2.28 | 0.002846 |
| 236029_at | FAT3 | 2.28 | 0.028061 |
| 1560916_a_at | DPY19L1 | 2.28 | 0.017852 |
| 1565906_at | NADSYN1 | 2.27 | 0.020126 |
| 1560509_at | NA | 2.27 | 0.027215 |
| 206289_at | HOXA4 | 2.27 | 0.004572 |
| 227697_at | SOCS3 | 2.27 | 0.010252 |
| 202510_s_at | TNFAIP2 | 2.27 | 0.010088 |
| 1564372_s_at | CASC2 | 2.26 | 0.031806 |
| 203388_at | ARRB2 | 2.26 | 0.010153 |
| 225735_at | ANKRD50 | 2.26 | 0.025666 |
| 226065_at | PRICKLE1 | 2.25 | 0.01555 |
| 206669_at | GAD1 | 2.25 | 0.007004 |
| 238139_at | NA | 2.25 | 0.005759 |
| 225078_at | EMP2 | 2.25 | 0.000615 |
| 224739_at | PIM3 | 2.25 | 0.000948 |
| 207843_x_at | CYB5A | 2.25 | 0.002581 |
| 219636_s_at | ARMC9 | 2.25 | 0.045322 |
| 233176_at | NA | 2.25 | 0.046197 |
| 227296_at | MFSD3 | 2.24 | 0.02048 |
| 232530_at | PLD1 | 2.24 | 0.006012 |
| 205677_s_at | DLEU1 | 2.24 | 0.004498 |
| 231880_at | FAM40B | 2.24 | 0.008159 |
| 225731_at | ANKRD50 | 2.24 | 0.020511 |
| 224604_at | C4orf3 | 2.24 | 0.027286 |
| 232322_x_at | STARD10 | 2.24 | 0.011897 |
| 217127_at | CTH | 2.23 | 0.040373 |
| 225822_at | TMEM125 | 2.23 | 0.002651 |
| 207808_s_at | PROS1 | 2.23 | 0.03578 |
| 236840_at | C12orf56 | 2.23 | 0.003298 |
| 232572_at | PCA3 | 2.23 | 0.01332 |
| 214614_at | MNX1 | 2.23 | 0.01075 |
| 1557346_a_at | NA | 2.23 | 0.028853 |
| 204778_x_at | HOXB7 | 2.22 | 0.03154 |
| 201764_at | TMEM106C | 2.22 | 0.000714 |
| 218309_at | CAMK2N1 | 2.21 | 0.02775 |
| 226487_at | C12orf34 | 2.21 | 0.003869 |
| 224681_at | GNA12 | 2.21 | 0.002505 |
| 222262_s_at | ETNK1 | 2.21 | 0.004601 |
| 227760_at | IGFBPL1 | 2.21 | 0.008192 |
| 204164_at | SIPA1 | 2.21 | 0.005645 |
| 206554_x_at | SETMAR | 2.21 | 0.036605 |
| 229014_at | FLJ42709 | 2.21 | 0.020341 |
| 223241_at | SNX8 | 2.20 | 0.035398 |
| 229072_at | NA | 2.20 | 0.003548 |
| 236471_at | NFE2L3 | 2.20 | 0.026903 |
| 224919_at | MRPS6 | 2.20 | 0.002275 |
| 209357_at | CITED2 | 2.19 | 0.024332 |
| 204646_at | DPYD | 2.19 | 0.004171 |
| 202598_at | S100A13 | 2.19 | 0.000931 |
| 203962_s_at | NEBL | 2.19 | 0.005883 |
| 230361_at | HEATR7A | 2.18 | 0.00269 |
| 219806_s_at | C11orf75 | 2.18 | 0.005223 |
| 202948_at | IL1R1 | 2.18 | 0.030788 |
| 210538_s_at | BIRC3 | 2.18 | 0.000637 |
| 228574_at | TMTC2 | 2.17 | 0.016529 |
| 221530_s_at | BHLHE41 | 2.17 | 0.000774 |
| 1566147_a_at | LOC375010 | 2.17 | 0.045978 |
| 210517_s_at | AKAP12 | 2.17 | 0.011904 |
| 200985_s_at | CD59 | 2.17 | 0.001054 |
| 225604_s_at | GLIPR2 | 2.16 | 0.006009 |
| 243629_x_at | NA | 2.16 | 0.019763 |
| 1558404_at | LOC644242 | 2.16 | 0.038576 |
| 1562484_at | FLJ35848 | 2.16 | 0.030071 |
| 225079_at | EMP2 | 2.16 | 0.002442 |
| 212996_s_at | URB1 | 2.16 | 0.007991 |
| 213940_s_at | FNBP1 | 2.16 | 0.013535 |
| 223103_at | STARD10 | 2.16 | 0.005226 |
| 219109_at | SPAG16 | 2.15 | 0.007432 |
| 206670_s_at | GAD1 | 2.15 | 0.006876 |
| 201211_s_at | DDX3X | 2.15 | 0.005756 |
| 224218_s_at | TRPS1 | 2.15 | 0.016717 |
| 218627_at | DRAM1 | 2.15 | 0.011704 |
| 232088_x_at | hCG_2039027 | 2.14 | 0.016666 |
| 230050_at | NACC2 | 2.14 | 0.013415 |
| 219342_at | CASD1 | 2.14 | 0.01728 |
| 225328_at | NA | 2.14 | 0.011229 |
| 209950_s_at | VILL | 2.14 | 0.022542 |
| 223594_at | TMEM117 | 2.14 | 0.011358 |
| 219736_at | TRIM36 | 2.13 | 0.032835 |
| 1558105_a_at | NA | 2.13 | 0.005454 |
| 241431_at | NA | 2.13 | 0.005962 |
| 201272_at | AKR1B1 | 2.13 | 0.000575 |
| 209101_at | CTGF | 2.13 | 0.04438 |
| 213164_at | SLC5A3 | 2.13 | 0.009009 |
| 204679_at | KCNK1 | 2.13 | 0.023237 |
| 219822_at | MTRF1 | 2.12 | 0.006362 |
| 218795_at | ACP6 | 2.12 | 0.004764 |
| 213109_at | TNIK | 2.12 | 0.016572 |
| 222736_s_at | TMEM38B | 2.11 | 0.00142 |
| 238590_x_at | TMEM107 | 2.11 | 0.002993 |
| 202016_at | MEST | 2.11 | 0.000818 |
| 212609_s_at | AKT3 | 2.11 | 0.042922 |
| 224463_s_at | C11orf70 | 2.11 | 0.020092 |
| 209710_at | GATA2 | 2.11 | 0.01034 |
| 204391_x_at | TRIM24 | 2.10 | 0.006195 |
| 227431_at | NA | 2.10 | 0.007987 |
| 203598_s_at | WBP4 | 2.10 | 0.005505 |
| 205618_at | PRRG1 | 2.09 | 0.001011 |
| 210827_s_at | ELF3 | 2.09 | 0.005873 |
| 226829_at | AFAP1L2 | 2.09 | 0.010396 |
| 244834_at | C1orf134 | 2.09 | 0.016542 |
| 235252_at | KSR1 | 2.09 | 0.025807 |
| 217999_s_at | PHLDA1 | 2.09 | 0.032252 |
| 227012_at | SLC25A40 | 2.09 | 0.004185 |
| 219690_at | TMEM149 | 2.08 | 0.007124 |
| 239785_at | DZIP1L | 2.08 | 0.032373 |
| 200897_s_at | PALLD | 2.08 | 0.012286 |
| 237435_at | NA | 2.08 | 0.04576 |
| 225282_at | SMAP2 | 2.08 | 0.002156 |
| 220952_s_at | PLEKHA5 | 2.07 | 0.01811 |
| 219017_at | ETNK1 | 2.07 | 0.007756 |
| 203781_at | MRPL33 | 2.07 | 0.029361 |
| 203058_s_at | PAPSS2 | 2.07 | 0.032827 |
| 239824_s_at | TMEM107 | 2.06 | 0.009815 |
| 225802_at | TOP1MT | 2.06 | 0.009526 |
| 218981_at | ACN9 | 2.06 | 0.017054 |
| 228600_x_at | C7orf46 | 2.06 | 0.011209 |
| 235134_at | NA | 2.06 | 0.010109 |
| 205075_at | SERPINF2 | 2.06 | 0.009121 |
| 219344_at | SLC29A3 | 2.06 | 0.009306 |
| 211123_at | SLC5A5 | 2.06 | 0.014806 |
| 238542_at | ULBP2 | 2.05 | 0.005131 |
| 235704_at | DAZAP2 | 2.05 | 0.015257 |
| 228654_at | SPIN4 | 2.05 | 0.007658 |
| 211548_s_at | HPGD | 2.05 | 0.026358 |
| 202457_s_at | PPP3CA | 2.05 | 0.001676 |
| 205608_s_at | ANGPT1 | 2.05 | 0.011121 |
| 1570153_at | C13orf38 | 2.05 | 0.039843 |
| 213327_s_at | USP12 | 2.05 | 0.001033 |
| 207446_at | TLR6 | 2.05 | 0.027576 |
| 203955_at | KIAA0649 | 2.05 | 0.009234 |
| 219911_s_at | SLCO4A1 | 2.05 | 0.031771 |
| 49679_s_at | EDEM2 | 2.05 | 0.021674 |
| 203158_s_at | GLS | 2.04 | 0.024024 |
| 1554119_at | C16orf57 | 2.04 | 0.015491 |
| 232113_at | NA | 2.04 | 0.030539 |
| 1558293_at | KIAA1107 | 2.04 | 0.038109 |
| 205716_at | SLC25A40 | 2.04 | 0.002708 |
| 219355_at | CXorf57 | 2.04 | 0.006227 |
| 40665_at | FMO3 | 2.04 | 0.018482 |
| 222634_s_at | TBL1XR1 | 2.03 | 0.035337 |
| 228661_s_at | NA | 2.03 | 0.037369 |
| 228765_at | GTF2IRD2 | 2.03 | 0.003183 |
| 226189_at | ITGB8 | 2.03 | 0.004384 |
| 218050_at | UFM1 | 2.03 | 0.004485 |
| 1557018_a_at | NA | 2.03 | 0.035374 |
| 226152_at | TTC7B | 2.03 | 0.006239 |
| 1553672_at | ENAH | 2.02 | 0.013185 |
| 209934_s_at | ATP2C1 | 2.02 | 0.024769 |
| 1053_at | RFC2 | 2.02 | 0.003628 |
| 201110_s_at | THBS1 | 2.02 | 0.013788 |
| 216010_x_at | FUT3 | 2.02 | 0.020233 |
| 203414_at | MMD | 2.02 | 0.015046 |
| 204867_at | GCHFR | 2.02 | 0.007441 |
| 204475_at | MMP1 | 2.02 | 0.011751 |
| 232594_at | LOC440498 | 2.01 | 0.006815 |
| 228405_at | RHPN1 | 2.01 | 0.026596 |
| 223878_at | INPP4B | 2.01 | 0.025207 |
| 240592_at | LCORL | 2.01 | 0.004541 |
| 237914_s_at | NA | 2.01 | 0.017158 |
| 214765_s_at | NAAA | 2.01 | 0.005596 |
| 222912_at | ARRB1 | 2.01 | 0.02863 |
| 1554264_at | CKAP2 | 2.01 | 0.026222 |
| 230696_at | NA | 2.01 | 0.013851 |
| 226604_at | TMTC3 | 2.00 | 0.004728 |

FDR: false discovery rate
