# Supplementary material for: Prediction of the Number of Activated Genes in Multiple Independent Cd+2- and As+3-Induced Malignant Transformations of Human Urothelial Cells (UROtsa)
Source: PLoS One. 2014 Jan 22;9(1):e85614. doi: 10.1371/journal.pone.0085614 (PMC3899011; doi:10.1371/journal.pone.0085614)
Supplement: Table S2 — The list of genes significantly repressed by Cadmium in Human Urothelial Cells. The table shows the Affymetrix probe IDs, the gene symbols, the fold changes and the false discovery rates. (DOCX) [file pone.0085614.s002.docx]

**Table S2**. The list of genes significantly repressed by Cadmium in Human Urothelial Cells.

| **Probe** | **Gene Symbol** | **Fold Change** | **FDR** |
| --- | --- | --- | --- |
| 200665_s_at | SPARC | 0.01 | 0.002819 |
| 204698_at | ISG20 | 0.03 | 7.96E-05 |
| 242625_at | RSAD2 | 0.03 | 0.002166 |
| 228575_at | IL20RB | 0.03 | 3.34E-05 |
| 227361_at | HS3ST3B1 | 0.04 | 0.00297 |
| 213797_at | RSAD2 | 0.04 | 0.001929 |
| 213122_at | TSPYL5 | 0.04 | 0.003928 |
| 205234_at | SLC16A4 | 0.05 | 0.001912 |
| 204830_x_at | PSG5 | 0.05 | 0.000465 |
| 211794_at | FYB | 0.05 | 0.002711 |
| 219593_at | SLC15A3 | 0.06 | 0.000441 |
| 231925_at | NA | 0.06 | 0.005368 |
| 202179_at | BLMH | 0.07 | 0.004604 |
| 222829_s_at | IL20RA | 0.08 | 0.00488 |
| 1553333_at | C1orf161 | 0.09 | 0.012253 |
| 1555854_at | NA | 0.09 | 0.000745 |
| 222760_at | ZNF703 | 0.10 | 0.000646 |
| 203980_at | FABP4 | 0.10 | 0.007389 |
| 230563_at | RASGEF1A | 0.10 | 0.00887 |
| 213240_s_at | KRT4 | 0.11 | 0.014056 |
| 225540_at | MAP2 | 0.11 | 0.03518 |
| 226757_at | IFIT2 | 0.11 | 0.002719 |
| 217502_at | IFIT2 | 0.12 | 0.001194 |
| 204614_at | SERPINB2 | 0.12 | 0.013541 |
| 204560_at | FKBP5 | 0.12 | 0.008223 |
| 232693_s_at | NA | 0.13 | 0.008159 |
| 208087_s_at | ZBP1 | 0.13 | 0.01275 |
| 235696_at | NA | 0.13 | 0.002396 |
| 219368_at | NAP1L2 | 0.13 | 0.03883 |
| 211741_x_at | PSG3 | 0.14 | 0.003652 |
| 238584_at | IQCA1 | 0.14 | 0.029904 |
| 235737_at | TSLP | 0.14 | 0.010489 |
| 235411_at | PGBD1 | 0.14 | 0.022129 |
| 204994_at | MX2 | 0.15 | 0.006758 |
| 222909_s_at | BAG4 | 0.15 | 0.016824 |
| 205421_at | SLC22A3 | 0.15 | 0.014939 |
| 226955_at | AFAP1L1 | 0.16 | 0.017161 |
| 216244_at | IL1RN | 0.16 | 0.048963 |
| 241871_at | CAMK4 | 0.17 | 0.049739 |
| 224148_at | FYB | 0.17 | 0.001001 |
| 203153_at | IFIT1 | 0.18 | 0.024817 |
| 218694_at | ARMCX1 | 0.18 | 0.000976 |
| 203888_at | THBD | 0.18 | 0.0278 |
| 206136_at | FZD5 | 0.18 | 0.014384 |
| 223741_s_at | TTYH2 | 0.18 | 0.017518 |
| 205285_s_at | FYB | 0.19 | 0.006528 |
| AFFX-HUMISGF3A/M97935_5_at | STAT1 | 0.19 | 0.011272 |
| 226702_at | CMPK2 | 0.19 | 0.02493 |
| 1567013_at | NFE2L2 | 0.20 | 0.00065 |
| 209699_x_at | AKR1C2 | 0.20 | 0.021386 |
| 202718_at | IGFBP2 | 0.20 | 0.029372 |
| 39549_at | NPAS2 | 0.20 | 0.036503 |
| 206133_at | XAF1 | 0.20 | 0.020515 |
| 202345_s_at | FABP5 | 0.20 | 0.000359 |
| 229427_at | SEMA5A | 0.20 | 0.007163 |
| 204747_at | IFIT3 | 0.20 | 0.015806 |
| 207455_at | P2RY1 | 0.20 | 0.00107 |
| 231867_at | ODZ2 | 0.20 | 0.000945 |
| 207849_at | IL2 | 0.21 | 0.0318 |
| 33304_at | ISG20 | 0.21 | 0.002994 |
| 203963_at | CA12 | 0.21 | 0.018751 |
| 209969_s_at | STAT1 | 0.21 | 0.007042 |
| 204533_at | CXCL10 | 0.21 | 0.00087 |
| 216926_s_at | KIAA0892 | 0.21 | 0.021158 |
| 201348_at | GPX3 | 0.22 | 0.03514 |
| 211653_x_at | AKR1C2 | 0.22 | 0.024072 |
| 219118_at | FKBP11 | 0.22 | 0.002437 |
| 225664_at | COL12A1 | 0.22 | 0.025647 |
| 223044_at | SLC40A1 | 0.22 | 0.038029 |
| 211122_s_at | CXCL11 | 0.22 | 0.001371 |
| 217626_at | NA | 0.23 | 0.004204 |
| 204948_s_at | FST | 0.23 | 0.000521 |
| 201367_s_at | ZFP36L2 | 0.23 | 0.007163 |
| 214091_s_at | GPX3 | 0.23 | 0.030455 |
| 203887_s_at | THBD | 0.23 | 0.006232 |
| 230275_at | ARSI | 0.23 | 0.00567 |
| 228620_at | NA | 0.23 | 0.023915 |
| 211795_s_at | FYB | 0.23 | 0.010222 |
| 1557924_s_at | ALPL | 0.23 | 0.018369 |
| 226847_at | FST | 0.23 | 0.000874 |
| 1562250_at | NA | 0.23 | 0.038256 |
| 210086_at | HR | 0.23 | 0.010415 |
| 209720_s_at | SERPINB3 | 0.24 | 0.018081 |
| 242277_at | NA | 0.24 | 0.001852 |
| 230036_at | SAMD9L | 0.24 | 0.017649 |
| 231577_s_at | GBP1 | 0.24 | 0.0229 |
| 205352_at | SERPINI1 | 0.24 | 0.018842 |
| 1553808_a_at | NKX2-3 | 0.24 | 0.039711 |
| 202869_at | OAS1 | 0.24 | 0.011381 |
| 229450_at | IFIT3 | 0.25 | 0.014563 |
| 209594_x_at | PSG9 | 0.25 | 0.000338 |
| 219209_at | IFIH1 | 0.25 | 0.008353 |
| 223316_at | CCDC3 | 0.25 | 0.009125 |
| 219684_at | RTP4 | 0.25 | 0.007669 |
| 231766_s_at | COL12A1 | 0.25 | 0.04092 |
| 216594_x_at | AKR1C1 | 0.25 | 0.037893 |
| 219352_at | HERC6 | 0.25 | 0.017573 |
| 210786_s_at | FLI1 | 0.25 | 0.026588 |
| 227266_s_at | FYB | 0.25 | 0.014319 |
| 203851_at | IGFBP6 | 0.26 | 0.013419 |
| 210505_at | ADH7 | 0.26 | 0.000107 |
| 1555464_at | IFIH1 | 0.26 | 0.029056 |
| 207345_at | FST | 0.26 | 0.001294 |
| 227662_at | SYNPO2 | 0.26 | 0.039678 |
| 225895_at | SYNPO2 | 0.26 | 0.008864 |
| 224724_at | SULF2 | 0.26 | 0.005424 |
| 204151_x_at | AKR1C1 | 0.26 | 0.028652 |
| 228439_at | BATF2 | 0.26 | 0.046101 |
| 210015_s_at | MAP2 | 0.26 | 0.025096 |
| 209792_s_at | KLK10 | 0.26 | 0.000882 |
| 1559776_at | GM2A | 0.26 | 0.002574 |
| 209719_x_at | SERPINB3 | 0.27 | 0.009403 |
| 228714_at | NA | 0.27 | 0.002913 |
| 227016_at | ERICH1 | 0.27 | 0.001205 |
| 205660_at | OASL | 0.27 | 0.0218 |
| 201481_s_at | PYGB | 0.27 | 0.025085 |
| AFFX-HUMISGF3A/M97935_MA_at | STAT1 | 0.27 | 0.010969 |
| 209587_at | PITX1 | 0.27 | 0.004034 |
| AFFX-HUMISGF3A/M97935_MB_at | STAT1 | 0.27 | 0.008638 |
| 224013_s_at | SOX7 | 0.27 | 0.002434 |
| 210163_at | CXCL11 | 0.27 | 0.007467 |
| 202270_at | GBP1 | 0.27 | 0.042986 |
| 202269_x_at | GBP1 | 0.27 | 0.018792 |
| 210655_s_at | NA | 0.28 | 0.011321 |
| 223468_s_at | RGMA | 0.28 | 0.002592 |
| 232958_at | NA | 0.28 | 0.027325 |
| 213169_at | SEMA5A | 0.28 | 0.001194 |
| 222895_s_at | BCL11B | 0.28 | 0.001431 |
| 227188_at | C21orf63 | 0.28 | 0.008404 |
| 233565_s_at | SDCBP2 | 0.28 | 0.003276 |
| 222717_at | SDPR | 0.28 | 0.002348 |
| 226625_at | TGFBR3 | 0.28 | 0.000881 |
| 229065_at | SLC35F3 | 0.28 | 0.023161 |
| 209488_s_at | RBPMS | 0.28 | 0.000984 |
| 209921_at | SLC7A11 | 0.29 | 0.001128 |
| 214422_at | RAD23B | 0.29 | 0.047923 |
| 218454_at | PLBD1 | 0.29 | 0.007404 |
| 1552648_a_at | TNFRSF10A | 0.29 | 0.016355 |
| 204070_at | RARRES3 | 0.29 | 0.001203 |
| 214059_at | IFI44 | 0.29 | 0.039547 |
| 1554703_at | ARHGEF10 | 0.30 | 0.039762 |
| 231775_at | TNFRSF10A | 0.30 | 0.000483 |
| 212739_s_at | NME4 | 0.30 | 0.019002 |
| 211319_at | RAE1 | 0.30 | 0.039869 |
| 218954_s_at | BRF2 | 0.30 | 0.010475 |
| 205552_s_at | OAS1 | 0.30 | 0.032728 |
| 206504_at | CYP24A1 | 0.30 | 0.018716 |
| 232654_s_at | NA | 0.30 | 0.004957 |
| 206237_s_at | NRG1 | 0.30 | 0.022039 |
| 204879_at | PDPN | 0.30 | 0.011519 |
| 222487_s_at | RPS27L | 0.30 | 0.002951 |
| 219091_s_at | MMRN2 | 0.30 | 0.001843 |
| 219277_s_at | OGDHL | 0.30 | 0.013896 |
| 238716_at | NA | 0.30 | 0.043646 |
| 1555330_at | GCLC | 0.31 | 0.030733 |
| 223360_at | C21orf56 | 0.31 | 0.031906 |
| 208891_at | DUSP6 | 0.31 | 0.000638 |
| 218943_s_at | DDX58 | 0.31 | 0.008863 |
| 206553_at | OAS2 | 0.31 | 0.005964 |
| 203343_at | UGDH | 0.31 | 0.006055 |
| 232417_x_at | ZDHHC11 | 0.31 | 0.049547 |
| 209738_x_at | PSG6 | 0.31 | 0.000748 |
| 209939_x_at | CFLAR | 0.31 | 0.001852 |
| 203009_at | BCAM | 0.31 | 0.020025 |
| 211317_s_at | CFLAR | 0.31 | 0.003573 |
| 212659_s_at | IL1RN | 0.31 | 0.012767 |
| 233555_s_at | SULF2 | 0.31 | 0.004719 |
| 203399_x_at | PSG3 | 0.31 | 0.010246 |
| 219269_at | HMBOX1 | 0.31 | 0.017053 |
| 219115_s_at | IL20RA | 0.32 | 0.000503 |
| 204972_at | OAS2 | 0.32 | 0.023963 |
| 208596_s_at | NA | 0.32 | 0.032178 |
| 202008_s_at | NID1 | 0.32 | 0.035744 |
| 212845_at | SAMD4A | 0.32 | 0.009693 |
| 221245_s_at | FZD5 | 0.32 | 0.001946 |
| 227062_at | NEAT1 | 0.32 | 0.028518 |
| 204952_at | LYPD3 | 0.32 | 0.001136 |
| 224225_s_at | ETV7 | 0.32 | 0.007931 |
| 231259_s_at | CCND2 | 0.33 | 0.010489 |
| 206094_x_at | NA | 0.33 | 0.01035 |
| 211834_s_at | TP63 | 0.33 | 0.004195 |
| 219301_s_at | CNTNAP2 | 0.33 | 0.017158 |
| 204047_s_at | PHACTR2 | 0.33 | 0.000775 |
| 227917_at | FAM85A | 0.33 | 0.000457 |
| 219117_s_at | FKBP11 | 0.33 | 0.00221 |
| 211588_s_at | NA | 0.33 | 0.005348 |
| 210754_s_at | LYN | 0.33 | 0.000507 |
| 204048_s_at | PHACTR2 | 0.33 | 0.000651 |
| 219856_at | C1orf116 | 0.33 | 0.003446 |
| 233504_at | C9orf84 | 0.33 | 0.001429 |
| 207126_x_at | NA | 0.33 | 0.01787 |
| 203585_at | ZNF185 | 0.33 | 0.019757 |
| 217199_s_at | STAT2 | 0.33 | 0.024076 |
| 232517_s_at | PRIC285 | 0.33 | 0.005358 |
| 211862_x_at | CFLAR | 0.34 | 0.002752 |
| 238692_at | BTBD11 | 0.34 | 0.008732 |
| 226279_at | PRSS23 | 0.34 | 0.001708 |
| 201369_s_at | ZFP36L2 | 0.34 | 0.004622 |
| 203596_s_at | IFIT5 | 0.34 | 0.033493 |
| 1552546_a_at | LETM2 | 0.34 | 0.005414 |
| 208502_s_at | PITX1 | 0.34 | 0.001886 |
| 228010_at | PPP2R2C | 0.34 | 0.000567 |
| 221187_s_at | FUZ | 0.34 | 0.015486 |
| 210797_s_at | OASL | 0.34 | 0.020728 |
| 203147_s_at | TRIM14 | 0.34 | 0.002128 |
| 207717_s_at | PKP2 | 0.34 | 0.001873 |
| 207836_s_at | RBPMS | 0.35 | 0.017592 |
| 238105_x_at | WNT7B | 0.35 | 0.009542 |
| 211883_x_at | CEACAM1 | 0.35 | 0.005396 |
| 212923_s_at | C6orf145 | 0.35 | 0.003464 |
| 40093_at | BCAM | 0.35 | 0.014345 |
| 215891_s_at | GM2A | 0.35 | 0.003912 |
| 216243_s_at | IL1RN | 0.35 | 0.014918 |
| 242961_x_at | DDX58 | 0.35 | 0.002882 |
| 241371_at | NA | 0.35 | 0.003602 |
| 204858_s_at | TYMP | 0.35 | 0.003427 |
| 227725_at | ST6GALNAC1 | 0.35 | 0.012243 |
| 218999_at | TMEM140 | 0.35 | 0.015937 |
| 221870_at | EHD2 | 0.35 | 0.006171 |
| 227475_at | FOXQ1 | 0.35 | 0.026411 |
| 228421_s_at | EFEMP1 | 0.35 | 0.009904 |
| 227642_at | TFCP2L1 | 0.35 | 0.048701 |
| 215783_s_at | ALPL | 0.36 | 0.01707 |
| 205623_at | ALDH3A1 | 0.36 | 0.006978 |
| 209611_s_at | SLC1A4 | 0.36 | 0.005227 |
| 221770_at | RPE | 0.36 | 0.001988 |
| 207788_s_at | SORBS3 | 0.36 | 0.023972 |
| 218149_s_at | ZNF395 | 0.36 | 0.000906 |
| 211195_s_at | TP63 | 0.36 | 0.003894 |
| 204731_at | TGFBR3 | 0.36 | 0.003525 |
| 212831_at | MEGF9 | 0.36 | 0.041423 |
| 239247_at | LOC401577 | 0.36 | 0.043643 |
| 204537_s_at | GABRE | 0.36 | 0.029451 |
| 219011_at | PLEKHA4 | 0.36 | 0.008006 |
| 232746_at | CXCR7 | 0.36 | 0.001313 |
| 209610_s_at | SLC1A4 | 0.36 | 0.001866 |
| 206424_at | CYP26A1 | 0.36 | 0.031399 |
| 205375_at | MDFI | 0.36 | 0.001058 |
| 244774_at | PHACTR2 | 0.36 | 0.010692 |
| 208485_x_at | CFLAR | 0.36 | 0.002181 |
| 215867_x_at | CA12 | 0.36 | 0.008119 |
| 223216_x_at | ZNF395 | 0.36 | 0.002773 |
| 219691_at | SAMD9 | 0.36 | 0.014107 |
| 223912_s_at | CLN8 | 0.36 | 0.004402 |
| 208230_s_at | NRG1 | 0.36 | 0.006876 |
| 205206_at | KAL1 | 0.37 | 0.003652 |
| 224996_at | ASPH | 0.37 | 0.003229 |
| 209556_at | NCDN | 0.37 | 0.004373 |
| 221898_at | PDPN | 0.37 | 0.022571 |
| 227070_at | GLT8D2 | 0.37 | 0.021584 |
| 223298_s_at | NT5C3 | 0.37 | 0.00538 |
| 219962_at | ACE2 | 0.37 | 0.009875 |
| 205241_at | SCO2 | 0.37 | 0.002103 |
| 203725_at | GADD45A | 0.37 | 0.004003 |
| 219211_at | USP18 | 0.37 | 0.022227 |
| 226109_at | C21orf91 | 0.37 | 0.000668 |
| 221123_x_at | ZNF395 | 0.37 | 0.004107 |
| 225076_s_at | ZNFX1 | 0.37 | 0.004703 |
| 202625_at | LYN | 0.37 | 0.002931 |
| 207043_s_at | SLC6A9 | 0.37 | 0.006221 |
| AFFX-HUMISGF3A/M97935_3_at | STAT1 | 0.37 | 0.012624 |
| 208012_x_at | SP110 | 0.37 | 0.002113 |
| 202626_s_at | LYN | 0.37 | 0.002024 |
| 204532_x_at | NA | 0.37 | 0.016727 |
| 222874_s_at | CLN8 | 0.37 | 0.000853 |
| 200887_s_at | STAT1 | 0.37 | 0.017096 |
| 225008_at | ASPH | 0.37 | 0.003471 |
| 205569_at | LAMP3 | 0.37 | 0.006538 |
| 208961_s_at | KLF6 | 0.37 | 0.001372 |
| 205170_at | STAT2 | 0.37 | 0.001355 |
| 205770_at | GSR | 0.38 | 0.00122 |
| 218400_at | OAS3 | 0.38 | 0.013539 |
| 209294_x_at | TNFRSF10B | 0.38 | 0.005627 |
| 214154_s_at | PKP2 | 0.38 | 0.014302 |
| 222793_at | DDX58 | 0.38 | 0.007367 |
| 215125_s_at | NA | 0.38 | 0.038822 |
| 227974_at | NA | 0.38 | 0.002948 |
| 208892_s_at | DUSP6 | 0.38 | 0.002578 |
| 204141_at | TUBB2A | 0.38 | 0.018486 |
| 237690_at | GPR115 | 0.38 | 0.018052 |
| 224989_at | NA | 0.38 | 0.026231 |
| 212657_s_at | IL1RN | 0.38 | 0.029248 |
| 236619_at | NA | 0.38 | 0.03805 |
| 212810_s_at | SLC1A4 | 0.38 | 0.001095 |
| 1552378_s_at | RDH10 | 0.38 | 0.023653 |
| 204049_s_at | PHACTR2 | 0.38 | 0.002145 |
| 209761_s_at | SP110 | 0.38 | 0.009399 |
| 209487_at | RBPMS | 0.38 | 0.002659 |
| 229303_at | NA | 0.38 | 0.002156 |
| 229638_at | IRX3 | 0.38 | 0.011101 |
| 225609_at | GSR | 0.38 | 0.00212 |
| 211552_s_at | ALDH4A1 | 0.38 | 0.00245 |
| 231274_s_at | NA | 0.38 | 0.007503 |
| 1562102_at | AKR1C1 | 0.38 | 0.0137 |
| 227102_at | TRIM35 | 0.39 | 0.005451 |
| 201957_at | PPP1R12B | 0.39 | 0.008755 |
| 209079_x_at | NA | 0.39 | 0.003381 |
| 201752_s_at | ADD3 | 0.39 | 0.003655 |
| 227816_at | NTN1 | 0.39 | 0.021679 |
| 1557236_at | APOL6 | 0.39 | 0.026497 |
| 201753_s_at | ADD3 | 0.39 | 0.006379 |
| 33646_g_at | GM2A | 0.39 | 0.002023 |
| 209385_s_at | PROSC | 0.39 | 0.00305 |
| 211929_at | HNRNPA3 | 0.39 | 0.004873 |
| 209135_at | ASPH | 0.39 | 0.000674 |
| 207713_s_at | RBCK1 | 0.39 | 0.000637 |
| 204249_s_at | LMO2 | 0.39 | 0.01001 |
| 238460_at | FAM83A | 0.39 | 0.02191 |
| 227947_at | PHACTR2 | 0.39 | 0.000734 |
| 223218_s_at | NFKBIZ | 0.39 | 0.006458 |
| 232666_at | OAS3 | 0.39 | 0.007316 |
| 223217_s_at | NFKBIZ | 0.39 | 0.009001 |
| 227899_at | VIT | 0.39 | 0.000548 |
| 205467_at | CASP10 | 0.39 | 0.015802 |
| 213274_s_at | CTSB | 0.40 | 0.0184 |
| 202755_s_at | GPC1 | 0.40 | 0.01953 |
| 223093_at | ANKH | 0.40 | 0.001197 |
| 239899_at | RNF145 | 0.40 | 0.042303 |
| 220941_s_at | C21orf91 | 0.40 | 0.000368 |
| 217681_at | WNT7B | 0.40 | 0.010572 |
| 203236_s_at | LGALS9 | 0.40 | 0.001878 |
| 237105_at | NA | 0.40 | 0.001881 |
| 227931_at | INO80D | 0.40 | 0.000695 |
| 202864_s_at | SP100 | 0.40 | 0.028605 |
| 209508_x_at | CFLAR | 0.40 | 0.010716 |
| 1553956_at | ALS2CR4 | 0.40 | 0.004559 |
| 213457_at | MFHAS1 | 0.40 | 0.01179 |
| 232375_at | NA | 0.40 | 0.00478 |
| 203595_s_at | IFIT5 | 0.40 | 0.035411 |
| 210735_s_at | CA12 | 0.40 | 0.017672 |
| 225040_s_at | RPE | 0.40 | 0.00414 |
| 212811_x_at | SLC1A4 | 0.40 | 0.000899 |
| 223094_s_at | ANKH | 0.40 | 0.001975 |
| 231034_s_at | NHSL1 | 0.40 | 0.00239 |
| 211316_x_at | CFLAR | 0.40 | 0.001312 |
| 216574_s_at | NA | 0.40 | 0.002613 |
| 224261_at | NA | 0.41 | 0.00667 |
| 218887_at | MRPL2 | 0.41 | 0.013111 |
| 203722_at | ALDH4A1 | 0.41 | 0.013383 |
| 220076_at | ANKH | 0.41 | 0.004286 |
| 206035_at | REL | 0.41 | 0.033621 |
| 225239_at | NA | 0.41 | 0.006502 |
| 201034_at | ADD3 | 0.41 | 0.004093 |
| 200879_s_at | EPAS1 | 0.41 | 0.016214 |
| 210218_s_at | SP100 | 0.41 | 0.046938 |
| 202067_s_at | LDLR | 0.41 | 0.00348 |
| 212876_at | B4GALT4 | 0.41 | 0.005374 |
| 208711_s_at | CCND1 | 0.41 | 0.009422 |
| 228607_at | OAS2 | 0.41 | 0.009458 |
| 221960_s_at | RAB2A | 0.41 | 0.013506 |
| 221920_s_at | SLC25A37 | 0.41 | 0.005991 |
| 1552727_s_at | ADAMTS17 | 0.41 | 0.012362 |
| 230314_at | NA | 0.41 | 0.008155 |
| 209365_s_at | ECM1 | 0.41 | 0.003818 |
| 242363_at | NA | 0.41 | 0.011928 |
| 213568_at | OSR2 | 0.41 | 0.010469 |
| 205882_x_at | ADD3 | 0.41 | 0.010049 |
| 208427_s_at | ELAVL2 | 0.41 | 0.004693 |
| 1558775_s_at | NSMAF | 0.41 | 0.017218 |
| 225510_at | OAF | 0.42 | 0.006705 |
| 210540_s_at | B4GALT4 | 0.42 | 0.000975 |
| 205574_x_at | BMP1 | 0.42 | 0.014342 |
| 235678_at | GM2A | 0.42 | 0.002956 |
| 210896_s_at | ASPH | 0.42 | 0.003693 |
| 209640_at | PML | 0.42 | 0.041129 |
| 201375_s_at | PPP2CB | 0.42 | 0.000349 |
| 217299_s_at | NBN | 0.42 | 0.002592 |
| 203110_at | PTK2B | 0.42 | 0.013579 |
| 202124_s_at | TRAK2 | 0.42 | 0.00947 |
| 228230_at | PRIC285 | 0.42 | 0.009405 |
| 214313_s_at | EIF5B | 0.42 | 0.011859 |
| 239067_s_at | PANX2 | 0.42 | 0.013252 |
| 39402_at | IL1B | 0.42 | 0.006668 |
| 218017_s_at | HGSNAT | 0.42 | 0.005184 |
| 222758_s_at | TMEM132A | 0.42 | 0.014281 |
| 223843_at | SCARA3 | 0.42 | 0.011997 |
| 211825_s_at | NA | 0.42 | 0.031766 |
| 211194_s_at | TP63 | 0.42 | 0.00675 |
| 225039_at | RPE | 0.42 | 0.000458 |
| 241869_at | APOL6 | 0.42 | 0.012751 |
| 209727_at | GM2A | 0.42 | 0.001584 |
| 220038_at | NA | 0.43 | 0.00853 |
| 242944_at | FAM83A | 0.43 | 0.015635 |
| 207595_s_at | BMP1 | 0.43 | 0.017658 |
| 206503_x_at | PML | 0.43 | 0.032458 |
| 205097_at | SLC26A2 | 0.43 | 0.021804 |
| 203180_at | ALDH1A3 | 0.43 | 0.013695 |
| 243271_at | NA | 0.43 | 0.027106 |
| 1556190_s_at | PRNP | 0.43 | 0.004797 |
| 225912_at | TP53INP1 | 0.43 | 0.003424 |
| 208436_s_at | IRF7 | 0.43 | 0.020263 |
| 209417_s_at | IFI35 | 0.43 | 0.016174 |
| 210563_x_at | CFLAR | 0.43 | 0.000668 |
| 243880_at | GOSR2 | 0.43 | 0.006586 |
| 228698_at | SOX7 | 0.43 | 0.000999 |
| 226863_at | FAM110C | 0.43 | 0.005459 |
| 207827_x_at | SNCA | 0.43 | 0.001183 |
| 210564_x_at | CFLAR | 0.43 | 0.037672 |
| 227963_at | NA | 0.43 | 0.025799 |
| 225840_at | TEF | 0.43 | 0.021633 |
| 221748_s_at | TNS1 | 0.43 | 0.011751 |
| 222699_s_at | PLEKHF2 | 0.43 | 0.000642 |
| 202922_at | GCLC | 0.43 | 0.007687 |
| 226275_at | MXD1 | 0.43 | 0.000956 |
| 228865_at | C1orf116 | 0.43 | 0.009125 |
| 224567_x_at | MALAT1 | 0.43 | 0.035944 |
| 223652_at | AS3MT | 0.43 | 0.014322 |
| 204466_s_at | SNCA | 0.43 | 0.014951 |
| 53720_at | C19orf66 | 0.43 | 0.008685 |
| 226778_at | C8orf42 | 0.43 | 0.027927 |
| 208106_x_at | PSG6 | 0.44 | 0.012288 |
| 211066_x_at | NA | 0.44 | 0.007123 |
| 201433_s_at | PTDSS1 | 0.44 | 0.000473 |
| 202350_s_at | MATN2 | 0.44 | 0.002726 |
| 1568592_at | TRIM69 | 0.44 | 0.049406 |
| 205732_s_at | NCOA2 | 0.44 | 0.002991 |
| 221087_s_at | APOL3 | 0.44 | 0.026359 |
| 204804_at | TRIM21 | 0.44 | 0.004416 |
| 225291_at | PNPT1 | 0.44 | 0.000758 |
| 1569157_s_at | ZNF846 | 0.44 | 0.014519 |
| 202827_s_at | MMP14 | 0.44 | 0.017327 |
| 224413_s_at | TM2D2 | 0.44 | 0.000454 |
| 209762_x_at | SP110 | 0.44 | 0.012604 |
| 1556026_at | NA | 0.44 | 0.04287 |
| 221447_s_at | GLT8D2 | 0.44 | 0.031564 |
| 202458_at | PRSS23 | 0.44 | 0.004105 |
| 225869_s_at | UNC93B1 | 0.44 | 0.001996 |
| 232263_at | SLC6A15 | 0.44 | 0.001834 |
| 210138_at | RGS20 | 0.44 | 0.00386 |
| 202895_s_at | SIRPA | 0.44 | 0.019196 |
| 226603_at | SAMD9L | 0.44 | 0.030941 |
| 224606_at | KLF6 | 0.44 | 0.004626 |
| 239370_at | NA | 0.44 | 0.034739 |
| 242387_at | C8orf42 | 0.44 | 0.023286 |
| 219165_at | PDLIM2 | 0.44 | 0.018483 |
| 227514_at | ITPRIPL2 | 0.45 | 0.029535 |
| 226144_at | REXO1 | 0.45 | 0.015361 |
| 230206_at | DOCK5 | 0.45 | 0.014746 |
| 216972_at | SPAM1 | 0.45 | 0.029134 |
| 217678_at | SLC7A11 | 0.45 | 0.005585 |
| 217437_s_at | TACC1 | 0.45 | 0.015092 |
| 212575_at | C19orf6 | 0.45 | 0.049636 |
| 210846_x_at | TRIM14 | 0.45 | 0.003323 |
| 202600_s_at | NRIP1 | 0.45 | 0.020905 |
| 1555832_s_at | KLF6 | 0.45 | 0.003342 |
| 230977_at | NPM2 | 0.45 | 0.01026 |
| 202906_s_at | NBN | 0.45 | 0.003178 |
| 202923_s_at | GCLC | 0.45 | 0.003435 |
| 201368_at | ZFP36L2 | 0.45 | 0.002447 |
| 234987_at | NA | 0.45 | 0.033273 |
| 1553322_s_at | TEAD1 | 0.45 | 0.045805 |
| 204451_at | FZD1 | 0.45 | 0.002648 |
| 224963_at | SLC26A2 | 0.45 | 0.005434 |
| 235638_at | RASSF6 | 0.45 | 0.01425 |
| 211889_x_at | CEACAM1 | 0.45 | 0.022843 |
| 201843_s_at | EFEMP1 | 0.45 | 0.006873 |
| 239151_at | RP11-144G6.7 | 0.45 | 0.014505 |
| 211013_x_at | PML | 0.45 | 0.018568 |
| 217173_s_at | LDLR | 0.45 | 0.012395 |
| 202701_at | BMP1 | 0.45 | 0.004874 |
| 218589_at | LPAR6 | 0.45 | 0.030741 |
| 205157_s_at | KRT17 | 0.46 | 0.012629 |
| 235292_at | FLJ32255 | 0.46 | 0.019448 |
| 204502_at | SAMHD1 | 0.46 | 0.011434 |
| 240201_at | NA | 0.46 | 0.048268 |
| 213976_at | CIZ1 | 0.46 | 0.003731 |
| 215046_at | C2orf67 | 0.46 | 0.03906 |
| 221542_s_at | ERLIN2 | 0.46 | 0.001545 |
| 202558_s_at | HSPA13 | 0.46 | 0.011914 |
| 204452_s_at | FZD1 | 0.46 | 0.005421 |
| 1564308_a_at | MPP7 | 0.46 | 0.004047 |
| 238346_s_at | TGS1 | 0.46 | 0.003071 |
| 210001_s_at | SOCS1 | 0.46 | 0.013763 |
| 200762_at | DPYSL2 | 0.46 | 0.009066 |
| 201668_x_at | MARCKS | 0.46 | 0.004301 |
| 221646_s_at | ZDHHC11 | 0.46 | 0.006581 |
| 203148_s_at | TRIM14 | 0.46 | 0.00925 |
| 218935_at | EHD3 | 0.46 | 0.000748 |
| 1552609_s_at | NA | 0.46 | 0.022032 |
| 207485_x_at | BTN3A1 | 0.46 | 0.024016 |
| 221045_s_at | PER3 | 0.46 | 0.008424 |
| 241418_at | LOC344887 | 0.46 | 0.001667 |
| 225046_at | NA | 0.47 | 0.010489 |
| 223980_s_at | SP110 | 0.47 | 0.002975 |
| 204467_s_at | SNCA | 0.47 | 0.035745 |
| 212093_s_at | MTUS1 | 0.47 | 0.00058 |
| 230193_at | WDR66 | 0.47 | 0.010716 |
| 1552283_s_at | ZDHHC11 | 0.47 | 0.011559 |
| 225062_at | LOC389831 | 0.47 | 0.0115 |
| 202907_s_at | NBN | 0.47 | 0.000525 |
| 226021_at | RDH10 | 0.47 | 0.021342 |
| 203410_at | AP3M2 | 0.47 | 0.007678 |
| 223577_x_at | MALAT1 | 0.47 | 0.010407 |
| 242953_at | ZNF234 | 0.47 | 0.022447 |
| 211272_s_at | DGKA | 0.47 | 0.005226 |
| 223385_at | CYP2S1 | 0.47 | 0.009923 |
| 233375_at | EFCAB2 | 0.47 | 0.023075 |
| 203790_s_at | HRSP12 | 0.47 | 0.000904 |
| 217279_x_at | MMP14 | 0.47 | 0.047858 |
| 227732_at | ATXN7L1 | 0.47 | 0.049033 |
| 209920_at | BMPR2 | 0.47 | 0.002287 |
| 219678_x_at | DCLRE1C | 0.47 | 0.003967 |
| 208712_at | CCND1 | 0.47 | 0.007124 |
| 1568868_at | CYP27C1 | 0.47 | 0.024152 |
| 224428_s_at | CDCA7 | 0.47 | 0.001837 |
| 204999_s_at | ATF5 | 0.47 | 0.042416 |
| 238451_at | MPP7 | 0.47 | 0.007819 |
| 222257_s_at | ACE2 | 0.47 | 0.02899 |
| 210405_x_at | TNFRSF10B | 0.47 | 0.005677 |
| 227105_at | CSPP1 | 0.47 | 0.001992 |
| 234989_at | NEAT1 | 0.47 | 0.00812 |
| 231068_at | SLC47A2 | 0.47 | 0.038394 |
| 202752_x_at | SLC7A8 | 0.48 | 0.029712 |
| 227717_at | FLJ41603 | 0.48 | 0.009489 |
| 207661_s_at | SH3PXD2A | 0.48 | 0.011018 |
| 226179_at | SLC25A37 | 0.48 | 0.006767 |
| 235964_x_at | NA | 0.48 | 0.044537 |
| 213294_at | EIF2AK2 | 0.48 | 0.025633 |
| 216674_at | HES2 | 0.48 | 0.014738 |
| 209253_at | SORBS3 | 0.48 | 0.010834 |
| 226936_at | C6orf173 | 0.48 | 0.011221 |
| 211607_x_at | EGFR | 0.48 | 0.045598 |
| 204433_s_at | SPATA2 | 0.48 | 0.008812 |
| 228260_at | ELAVL2 | 0.48 | 0.009024 |
| 218711_s_at | SDPR | 0.48 | 0.014693 |
| 217025_s_at | DBN1 | 0.48 | 0.013478 |
| 223167_s_at | USP25 | 0.48 | 0.014919 |
| 211546_x_at | SNCA | 0.48 | 0.01885 |
| 227829_at | GYLTL1B | 0.48 | 0.027297 |
| 224916_at | TMEM173 | 0.48 | 0.011208 |
| 235529_x_at | NA | 0.48 | 0.026904 |
| 238496_at | NA | 0.49 | 0.013006 |
| 1554791_a_at | C2orf67 | 0.49 | 0.007108 |
| 209384_at | PROSC | 0.49 | 0.002506 |
| 219468_s_at | CUEDC1 | 0.49 | 0.048061 |
| 230411_at | NA | 0.49 | 0.001268 |
| 205470_s_at | KLK11 | 0.49 | 0.017006 |
| 218027_at | MRPL15 | 0.49 | 0.000627 |
| 205717_x_at | NA | 0.49 | 0.002653 |
| 201469_s_at | SHC1 | 0.49 | 0.003867 |
| 226959_at | LOC283070 | 0.49 | 0.005283 |
| 221978_at | HLA-F | 0.49 | 0.04468 |
| 1553031_at | GPR115 | 0.49 | 0.003405 |
| 207382_at | TP63 | 0.49 | 0.004841 |
| 215836_s_at | NA | 0.49 | 0.014251 |
| 208003_s_at | NFAT5 | 0.49 | 0.03081 |
| 205667_at | WRN | 0.49 | 0.001433 |
| 230207_s_at | DOCK5 | 0.49 | 0.019824 |
| 207722_s_at | BTBD2 | 0.49 | 0.040888 |
| 231874_at | FAM126B | 0.49 | 0.007368 |
| 215983_s_at | UBXN8 | 0.49 | 0.009314 |
| 238741_at | FAM83A | 0.49 | 0.009205 |
| 227627_at | SGK3 | 0.49 | 0.007448 |
| 223110_at | KIAA1429 | 0.49 | 0.005153 |
| 214545_s_at | PROSC | 0.49 | 0.004636 |
| 211051_s_at | EXTL3 | 0.49 | 0.007659 |
| 229283_at | LOC728613 | 0.49 | 0.005103 |
| 219340_s_at | CLN8 | 0.49 | 0.008405 |
| 230903_s_at | C8orf42 | 0.49 | 0.02254 |
| 218250_s_at | CNOT7 | 0.49 | 0.002362 |
| 228168_at | ATP5G3 | 0.49 | 0.019777 |
| 222233_s_at | DCLRE1C | 0.49 | 0.001889 |
| 225186_at | RAPH1 | 0.49 | 0.044251 |
| 209750_at | NR1D2 | 0.49 | 0.02022 |
| 232382_s_at | PCMTD1 | 0.49 | 0.026979 |
| 213243_at | VPS13B | 0.49 | 0.01817 |
| 204505_s_at | EPB49 | 0.49 | 0.002373 |
| 214011_s_at | NOP16 | 0.49 | 0.012373 |
| 244838_at | NA | 0.49 | 0.034822 |
| 210057_at | SMG1 | 0.50 | 0.049166 |
| 210362_x_at | PML | 0.50 | 0.022747 |
| 1559477_s_at | MEIS1 | 0.50 | 0.022797 |
| 206008_at | TGM1 | 0.50 | 0.029829 |
| 203173_s_at | C16orf62 | 0.50 | 0.016571 |
| 225902_at | NA | 0.50 | 0.007012 |
| 235085_at | PRAGMIN | 0.50 | 0.000774 |
| 209119_x_at | NR2F2 | 0.50 | 0.024227 |
| 229548_at | NA | 0.50 | 0.023121 |
| 228007_at | C6orf204 | 0.50 | 0.037166 |

FDR: false discovery rate
