# Supplementary material for: Prediction of the Number of Activated Genes in Multiple Independent Cd+2- and As+3-Induced Malignant Transformations of Human Urothelial Cells (UROtsa)
Source: PLoS One. 2014 Jan 22;9(1):e85614. doi: 10.1371/journal.pone.0085614 (PMC3899011; doi:10.1371/journal.pone.0085614)
Supplement: Table S3 — The list of genes significantly induced by Arsenic in Human Urothelial Cells. The table shows the Affymetrix probe IDs, the gene symbols, the fold changes and the false discovery rates. (DOCX) [file pone.0085614.s003.docx]

**Table S3**. The list of genes significantly induced by Arsenic in Human Urothelial Cells.

| **Probe** | **Gene Symbol** | **Fold Change** | **FDR** |
| --- | --- | --- | --- |
| 203440_at | CDH2 | 56.90 | 0.012079 |
| 208358_s_at | UGT8 | 45.07 | 0.000393 |
| 228956_at | UGT8 | 36.56 | 0.009989 |
| 206953_s_at | LPHN2 | 32.19 | 0.049925 |
| 1552511_a_at | CPA6 | 32.14 | 0.000104 |
| 219274_at | TSPAN12 | 26.27 | 0.03118 |
| 212328_at | LIMCH1 | 23.72 | 0.02209 |
| 209505_at | NR2F1 | 22.64 | 0.049776 |
| 226875_at | DOCK11 | 18.51 | 0.013685 |
| 227370_at | FAM171B | 18.10 | 0.034616 |
| 234980_at | TMEM56 | 15.85 | 0.003857 |
| 209267_s_at | SLC39A8 | 15.52 | 0.012756 |
| 222146_s_at | TCF4 | 15.31 | 0.038558 |
| 228066_at | C17orf96 | 13.97 | 0.025168 |
| 219983_at | HRASLS | 13.89 | 0.003036 |
| 217818_s_at | ARPC4 | 12.49 | 0.014488 |
| 237806_s_at | LOC729296 | 12.13 | 0.042908 |
| 219984_s_at | HRASLS | 10.67 | 0.013629 |
| 1561663_at | NA | 10.50 | 0.007259 |
| 1554533_at | C2 | 10.32 | 0.023601 |
| 230964_at | FREM2 | 10.17 | 0.045253 |
| 226864_at | PKIA | 9.83 | 0.030297 |
| 221004_s_at | ITM2C | 9.37 | 0.024249 |
| 202669_s_at | EFNB2 | 9.34 | 0.026638 |
| 1564794_at | NA | 8.63 | 0.046611 |
| 203441_s_at | CDH2 | 8.60 | 0.03176 |
| 209278_s_at | TFPI2 | 8.46 | 0.042771 |
| 207363_at | RS1 | 8.43 | 0.013863 |
| 201279_s_at | DAB2 | 8.38 | 0.036747 |
| 205593_s_at | PDE9A | 8.03 | 0.049734 |
| 242939_at | TFDP1 | 7.95 | 0.040249 |
| 227176_at | SLC2A13 | 7.62 | 0.025296 |
| 202668_at | EFNB2 | 7.61 | 0.030807 |
| 226459_at | PIK3AP1 | 7.39 | 0.037928 |
| 227209_at | CNTN1 | 7.22 | 0.015472 |
| 238029_s_at | SLC16A14 | 7.17 | 0.018869 |
| 230710_at | NA | 7.17 | 0.023118 |
| 208253_at | SIGLEC8 | 7.12 | 0.034822 |
| 222847_s_at | EGLN3 | 7.10 | 0.049122 |
| 232235_at | DSEL | 6.92 | 0.023099 |
| 219683_at | FZD3 | 6.82 | 0.044761 |
| 210415_s_at | ODF2 | 6.72 | 0.021496 |
| 242573_at | NA | 6.63 | 0.029883 |
| 238155_at | NA | 6.44 | 0.023384 |
| 203488_at | LPHN1 | 6.41 | 0.032045 |
| 209652_s_at | PGF | 6.34 | 0.044353 |
| 222062_at | IL27RA | 5.81 | 0.013624 |
| 215446_s_at | LOX | 5.71 | 0.016013 |
| 204298_s_at | LOX | 5.64 | 0.012911 |
| 210757_x_at | DAB2 | 5.53 | 0.019838 |
| 202478_at | TRIB2 | 5.52 | 0.026194 |
| 223642_at | ZIC2 | 5.50 | 0.031533 |
| 229800_at | DCLK1 | 5.49 | 0.020676 |
| 225971_at | DDHD1 | 5.35 | 0.013745 |
| 207076_s_at | ASS1 | 5.29 | 0.034792 |
| 203753_at | TCF4 | 5.28 | 0.032555 |
| 219373_at | DPM3 | 5.21 | 0.014174 |
| 214297_at | CSPG4 | 5.12 | 0.035554 |
| 205141_at | ANG | 5.06 | 0.03467 |
| 227782_at | ZBTB7C | 5.03 | 0.048877 |
| 232481_s_at | SLITRK6 | 4.98 | 0.039789 |
| 219295_s_at | PCOLCE2 | 4.97 | 0.028314 |
| 209909_s_at | TGFB2 | 4.90 | 0.042301 |
| 235371_at | GLT8D4 | 4.71 | 0.027262 |
| 226069_at | PRICKLE1 | 4.62 | 0.019355 |
| 213943_at | TWIST1 | 4.62 | 0.020205 |
| 200762_at | DPYSL2 | 4.56 | 0.010788 |
| 224870_at | KIAA0114 | 4.55 | 0.04475 |
| 237034_at | NA | 4.52 | 0.024978 |
| 219869_s_at | SLC39A8 | 4.52 | 0.049725 |
| 234996_at | CALCRL | 4.33 | 0.029416 |
| 219806_s_at | C11orf75 | 4.16 | 0.023248 |
| 213587_s_at | ATP6V0E2 | 4.08 | 0.016748 |
| 230110_at | MCOLN2 | 4.06 | 0.049768 |
| 232760_at | TEX15 | 3.98 | 0.012383 |
| 227296_at | MFSD3 | 3.96 | 0.040503 |
| 228551_at | DENND5B | 3.95 | 0.021777 |
| 205968_at | KCNS3 | 3.87 | 0.036041 |
| 228889_at | C14orf128 | 3.87 | 0.048554 |
| 212944_at | SLC5A3 | 3.79 | 0.021068 |
| 203157_s_at | GLS | 3.79 | 0.042457 |
| 227063_at | C17orf61 | 3.79 | 0.018949 |
| 223999_at | PPIL2 | 3.77 | 0.043337 |
| 1561017_at | NA | 3.66 | 0.031868 |
| 226487_at | C12orf34 | 3.65 | 0.03184 |
| 204798_at | MYB | 3.60 | 0.039836 |
| 235134_at | NA | 3.60 | 0.01324 |
| 205055_at | ITGAE | 3.59 | 0.010011 |
| 228762_at | LFNG | 3.58 | 0.043263 |
| 1556244_s_at | LOC375196 | 3.57 | 0.020253 |
| 222857_s_at | KCNMB4 | 3.54 | 0.014553 |
| 1560509_at | NA | 3.50 | 0.04297 |
| 240382_at | NA | 3.49 | 0.042974 |
| 1558501_at | DNM3 | 3.49 | 0.047805 |
| 238058_at | LOC150381 | 3.47 | 0.04792 |
| 206670_s_at | GAD1 | 3.44 | 0.048188 |
| 206669_at | GAD1 | 3.43 | 0.047994 |
| 225967_s_at | C17orf89 | 3.40 | 0.008002 |
| 210633_x_at | KRT10 | 3.37 | 0.021498 |
| 208237_x_at | ADAM22 | 3.34 | 0.031372 |
| 225670_at | FAM173B | 3.32 | 0.034767 |
| 230465_at | HS2ST1 | 3.28 | 0.025926 |
| 223172_s_at | MTP18 | 3.26 | 0.015924 |
| 40020_at | CELSR3 | 3.23 | 0.025974 |
| 224919_at | MRPS6 | 3.17 | 0.033605 |
| 200907_s_at | PALLD | 3.17 | 0.028195 |
| 213338_at | TMEM158 | 3.16 | 0.03831 |
| 219911_s_at | SLCO4A1 | 3.15 | 0.043498 |
| 214742_at | AZI1 | 3.14 | 0.020441 |
| 229860_x_at | C4orf48 | 3.12 | 0.015135 |
| 201718_s_at | EPB41L2 | 3.11 | 0.03567 |
| 225142_at | JHDM1D | 3.08 | 0.021394 |
| 228087_at | CCDC126 | 3.07 | 0.025319 |
| 219690_at | TMEM149 | 3.05 | 0.042076 |
| 224637_at | LOC100128731 | 3.03 | 0.017077 |
| 231396_s_at | FAM126A | 3.02 | 0.045004 |
| 239896_at | NA | 3.00 | 0.039257 |
| 213508_at | C14orf147 | 3.00 | 0.043961 |
| 229581_at | ELFN1 | 2.98 | 0.049839 |
| 218529_at | CD320 | 2.98 | 0.023179 |
| 229295_at | LOC150166 | 2.96 | 0.041759 |
| 211753_s_at | RLN1 | 2.95 | 0.02305 |
| 221791_s_at | CCDC72 | 2.95 | 0.012843 |
| 226931_at | TMTC1 | 2.93 | 0.039571 |
| 228972_at | NA | 2.93 | 0.020695 |
| 235466_s_at | DISP1 | 2.92 | 0.047091 |
| 228899_at | LOC100132884 | 2.92 | 0.020412 |
| 38671_at | PLXND1 | 2.92 | 0.038505 |
| 235299_at | SLC41A2 | 2.90 | 0.046133 |
| 229014_at | FLJ42709 | 2.90 | 0.047386 |
| 218274_s_at | ANKZF1 | 2.90 | 0.043753 |
| 1561906_at | NA | 2.88 | 0.045474 |
| 211602_s_at | TRPC1 | 2.88 | 0.036758 |
| 228061_at | CCDC126 | 2.88 | 0.048609 |
| 219736_at | TRIM36 | 2.88 | 0.043625 |
| 232322_x_at | STARD10 | 2.85 | 0.043213 |
| 230708_at | PRICKLE1 | 2.83 | 0.046965 |
| 230706_s_at | CAMK2N2 | 2.83 | 0.020273 |
| 207023_x_at | KRT10 | 2.81 | 0.022149 |
| 235191_at | LOC148189 | 2.79 | 0.033486 |
| 211709_s_at | CLEC11A | 2.74 | 0.036096 |
| 203058_s_at | PAPSS2 | 2.72 | 0.038193 |
| 239082_at | NA | 2.71 | 0.014513 |
| 222530_s_at | MKKS | 2.70 | 0.023661 |
| 232914_s_at | SYTL2 | 2.70 | 0.018657 |
| 225800_at | JAZF1 | 2.69 | 0.035588 |
| 213287_s_at | KRT10 | 2.67 | 0.044128 |
| 235704_at | DAZAP2 | 2.66 | 0.032864 |
| 203878_s_at | MMP11 | 2.65 | 0.049706 |
| 227282_at | PCDH19 | 2.65 | 0.044991 |
| 233587_s_at | SIPA1L2 | 2.64 | 0.040554 |
| 224604_at | C4orf3 | 2.64 | 0.040168 |
| 216643_at | NA | 2.63 | 0.034667 |
| 205777_at | DUSP9 | 2.63 | 0.033431 |
| 201848_s_at | BNIP3 | 2.63 | 0.031382 |
| 227479_at | KIAA1244 | 2.61 | 0.027376 |
| 201533_at | CTNNB1 | 2.61 | 0.036641 |
| 208308_s_at | GPI | 2.57 | 0.042311 |
| 227233_at | TSPAN2 | 2.55 | 0.031102 |
| 217127_at | CTH | 2.54 | 0.029713 |
| 219419_at | C18orf22 | 2.54 | 0.034228 |
| 231733_at | CARD18 | 2.54 | 0.036544 |
| 203484_at | SEC61G | 2.53 | 0.029068 |
| 222582_at | PRKAG2 | 2.53 | 0.037968 |
| 210130_s_at | TM7SF2 | 2.52 | 0.038929 |
| 202655_at | MANF | 2.50 | 0.018184 |
| 218718_at | PDGFC | 2.49 | 0.037431 |
| 218741_at | CENPM | 2.49 | 0.034262 |
| 240574_at | NA | 2.49 | 0.038197 |
| 230696_at | NA | 2.48 | 0.047695 |
| 213424_at | KIAA0895 | 2.48 | 0.027283 |
| 209605_at | TST | 2.48 | 0.023218 |
| 201105_at | LGALS1 | 2.47 | 0.044645 |
| 207351_s_at | SH2D2A | 2.45 | 0.03311 |
| 1556111_s_at | NA | 2.44 | 0.033894 |
| 226121_at | DHRS13 | 2.44 | 0.043285 |
| 232251_at | NUDT16P | 2.44 | 0.047613 |
| 228933_at | NHS | 2.42 | 0.026043 |
| 201397_at | PHGDH | 2.42 | 0.045767 |
| 235010_at | LOC729013 | 2.39 | 0.031871 |
| 241937_s_at | WDR4 | 2.37 | 0.047302 |
| 206376_at | SLC6A15 | 2.36 | 0.021204 |
| 200897_s_at | PALLD | 2.33 | 0.021167 |
| 224575_at | C3orf10 | 2.33 | 0.026607 |
| 203272_s_at | TUSC2 | 2.32 | 0.025267 |
| 223546_x_at | LUC7L | 2.31 | 0.030591 |
| 205443_at | SNAPC1 | 2.31 | 0.046045 |
| 228730_s_at | SCRN2 | 2.31 | 0.049337 |
| 241816_at | C14orf106 | 2.30 | 0.044485 |
| 209445_x_at | C7orf44 | 2.30 | 0.043612 |
| 240616_at | NA | 2.29 | 0.031973 |
| 202671_s_at | PDXK | 2.28 | 0.031834 |
| 1569107_s_at | ZNF642 | 2.27 | 0.049562 |
| 207071_s_at | ACO1 | 2.26 | 0.049242 |
| 226336_at | PPIA | 2.25 | 0.038088 |
| 201892_s_at | IMPDH2 | 2.25 | 0.036927 |
| 202749_at | WRB | 2.24 | 0.03152 |
| 208091_s_at | VOPP1 | 2.24 | 0.046883 |
| 211730_s_at | POLR2L | 2.24 | 0.026199 |
| 226882_x_at | WDR4 | 2.22 | 0.032627 |
| 232455_x_at | NA | 2.22 | 0.034826 |
| 204612_at | PKIA | 2.19 | 0.041792 |
| 226473_at | CBX2 | 2.18 | 0.048379 |
| 216588_at | NA | 2.18 | 0.034012 |
| 212345_s_at | CREB3L2 | 2.18 | 0.04822 |
| 219883_at | C11orf20 | 2.17 | 0.047105 |
| 212694_s_at | PCCB | 2.17 | 0.042158 |
| 202460_s_at | LPIN2 | 2.17 | 0.036666 |
| 225044_at | NT5C3L | 2.12 | 0.049998 |
| 229453_at | NA | 2.12 | 0.034814 |
| 218283_at | SS18L2 | 2.10 | 0.024832 |
| 243750_x_at | C21orf70 | 2.09 | 0.044676 |
| 226004_at | CABLES2 | 2.09 | 0.047909 |
| 228660_x_at | SEMA4F | 2.09 | 0.031926 |
| 207843_x_at | CYB5A | 2.09 | 0.046473 |
| 219109_at | SPAG16 | 2.06 | 0.031473 |
| 226193_x_at | NA | 2.06 | 0.032263 |
| 205531_s_at | GLS2 | 2.06 | 0.031331 |
| 203228_at | PAFAH1B3 | 2.05 | 0.037736 |
| 215984_s_at | ARFRP1 | 2.05 | 0.038711 |
| 210534_s_at | B9D1 | 2.04 | 0.042795 |
| 227442_at | COX18 | 2.03 | 0.047138 |
| 218153_at | CARS2 | 2.02 | 0.046125 |
| 227068_at | PGK1 | 2.02 | 0.031939 |
| 203945_at | ARG2 | 2.01 | 0.044117 |
| 225743_at | RPUSD3 | 2.01 | 0.044288 |
| 204766_s_at | NUDT1 | 2.01 | 0.048719 |
| 222634_s_at | TBL1XR1 | 2.00 | 0.043717 |

FDR: false discovery rate
