# Supplementary material for: Prediction of the Number of Activated Genes in Multiple Independent Cd+2- and As+3-Induced Malignant Transformations of Human Urothelial Cells (UROtsa)
Source: PLoS One. 2014 Jan 22;9(1):e85614. doi: 10.1371/journal.pone.0085614 (PMC3899011; doi:10.1371/journal.pone.0085614)
Supplement: Table S4 — The list of genes significantly repressed by Arsenic in Human Urothelial Cells. The table shows the Affymetrix probe IDs, the gene symbols, the fold changes and the false discovery rates. (DOCX) [file pone.0085614.s004.docx]

**Table S4**. The list of genes significantly repressed by Arsenic in Human Urothelial Cells.

| **Probe** | **Gene Symbol** | **Fold Change** | **FDR** |
| --- | --- | --- | --- |
| 200665_s_at | SPARC | 0.00 | 0.031504 |
| 204830_x_at | PSG5 | 0.02 | 0.012701 |
| 204614_at | SERPINB2 | 0.06 | 0.045235 |
| 204895_x_at | MUC4 | 0.07 | 0.048772 |
| 211794_at | FYB | 0.07 | 0.037612 |
| 217109_at | MUC4 | 0.07 | 0.049993 |
| 204777_s_at | MAL | 0.09 | 0.037206 |
| 208087_s_at | ZBP1 | 0.14 | 0.040418 |
| 219352_at | HERC6 | 0.15 | 0.036533 |
| 201367_s_at | ZFP36L2 | 0.16 | 0.028207 |
| 224225_s_at | ETV7 | 0.18 | 0.049082 |
| 202357_s_at | NA | 0.20 | 0.033287 |
| 209488_s_at | RBPMS | 0.20 | 0.039769 |
| 212659_s_at | IL1RN | 0.21 | 0.047484 |
| 215092_s_at | NFAT5 | 0.22 | 0.049892 |
| 216926_s_at | KIAA0892 | 0.22 | 0.048419 |
| 215342_s_at | RABGAP1L | 0.22 | 0.016985 |
| 225540_at | MAP2 | 0.23 | 0.044472 |
| 203963_at | CA12 | 0.23 | 0.023285 |
| 33304_at | ISG20 | 0.24 | 0.047593 |
| 211317_s_at | CFLAR | 0.24 | 0.031819 |
| 207043_s_at | SLC6A9 | 0.25 | 0.048566 |
| 215073_s_at | NR2F2 | 0.25 | 0.036902 |
| 216243_s_at | IL1RN | 0.25 | 0.042415 |
| 217626_at | NA | 0.26 | 0.033635 |
| 213982_s_at | RABGAP1L | 0.26 | 0.007672 |
| 203888_at | THBD | 0.27 | 0.03471 |
| 203887_s_at | THBD | 0.27 | 0.046718 |
| 209939_x_at | CFLAR | 0.28 | 0.045967 |
| 231879_at | COL12A1 | 0.28 | 0.032901 |
| 225814_at | XRN1 | 0.28 | 0.025282 |
| 212923_s_at | C6orf145 | 0.28 | 0.013664 |
| 39249_at | AQP3 | 0.28 | 0.031442 |
| 211530_x_at | HLA-G | 0.29 | 0.024455 |
| 235073_at | SCAMP4 | 0.29 | 0.038927 |
| 225957_at | C5orf41 | 0.29 | 0.042739 |
| 235016_at | REEP3 | 0.30 | 0.023379 |
| 201957_at | PPP1R12B | 0.30 | 0.03457 |
| 212067_s_at | C1R | 0.30 | 0.037293 |
| 230193_at | WDR66 | 0.31 | 0.044664 |
| 209610_s_at | SLC1A4 | 0.31 | 0.023396 |
| 212303_x_at | KHSRP | 0.32 | 0.04994 |
| 212336_at | EPB41L1 | 0.33 | 0.048545 |
| 231766_s_at | COL12A1 | 0.33 | 0.049904 |
| 235337_at | NA | 0.33 | 0.028664 |
| 208485_x_at | CFLAR | 0.33 | 0.028415 |
| 210540_s_at | B4GALT4 | 0.33 | 0.042615 |
| 203020_at | RABGAP1L | 0.33 | 0.036592 |
| 225510_at | OAF | 0.33 | 0.03681 |
| 231956_at | KIAA1618 | 0.34 | 0.047686 |
| 211862_x_at | CFLAR | 0.35 | 0.038563 |
| 227947_at | PHACTR2 | 0.35 | 0.044759 |
| 212262_at | QKI | 0.36 | 0.039826 |
| 211528_x_at | HLA-G | 0.36 | 0.046186 |
| 217497_at | TYMP | 0.37 | 0.034585 |
| 209750_at | NR1D2 | 0.37 | 0.016476 |
| 210563_x_at | CFLAR | 0.38 | 0.036379 |
| 236620_at | RIF1 | 0.38 | 0.019434 |
| 221766_s_at | FAM46A | 0.38 | 0.048366 |
| 219856_at | C1orf116 | 0.38 | 0.028377 |
| 222196_at | LOC286434 | 0.38 | 0.036462 |
| 208711_s_at | CCND1 | 0.39 | 0.01379 |
| 225956_at | C5orf41 | 0.39 | 0.03754 |
| 36564_at | RNF19B | 0.39 | 0.036823 |
| 217924_at | C6orf106 | 0.39 | 0.027247 |
| 1569269_s_at | SRGAP1 | 0.39 | 0.04882 |
| 207194_s_at | ICAM4 | 0.39 | 0.03804 |
| 238449_at | LOC595101 | 0.39 | 0.040113 |
| 235892_at | NA | 0.39 | 0.04525 |
| 215867_x_at | CA12 | 0.40 | 0.044829 |
| 226275_at | MXD1 | 0.40 | 0.046633 |
| 211799_x_at | HLA-C | 0.40 | 0.040154 |
| 220740_s_at | SLC12A6 | 0.40 | 0.028605 |
| 233888_s_at | SRGAP1 | 0.41 | 0.035664 |
| 214543_x_at | QKI | 0.41 | 0.021075 |
| 210514_x_at | HLA-G | 0.41 | 0.033408 |
| 212811_x_at | SLC1A4 | 0.42 | 0.041194 |
| 227931_at | INO80D | 0.42 | 0.034376 |
| 234969_s_at | EPC1 | 0.42 | 0.019796 |
| 203610_s_at | TRIM38 | 0.42 | 0.032286 |
| 226144_at | REXO1 | 0.42 | 0.041862 |
| 1558028_x_at | LOC647979 | 0.43 | 0.042619 |
| 1554830_a_at | STEAP3 | 0.43 | 0.044047 |
| 223144_s_at | AKIRIN2 | 0.43 | 0.035414 |
| 1561775_at | NA | 0.43 | 0.041328 |
| 223596_at | SLC12A6 | 0.44 | 0.034356 |
| 211014_s_at | NA | 0.44 | 0.022986 |
| 233917_s_at | MOV10 | 0.44 | 0.024871 |
| 204647_at | HOMER3 | 0.44 | 0.044346 |
| 212179_at | SFRS18 | 0.44 | 0.032389 |
| 209454_s_at | TEAD3 | 0.45 | 0.021443 |
| 233632_s_at | XRN1 | 0.45 | 0.030541 |
| 1554648_a_at | DUOXA1 | 0.45 | 0.033306 |
| 215836_s_at | NA | 0.46 | 0.025844 |
| 202495_at | TBCC | 0.46 | 0.02484 |
| 225181_at | ARID1B | 0.46 | 0.048918 |
| 225417_at | EPC1 | 0.47 | 0.045052 |
| 203427_at | ASF1A | 0.47 | 0.038192 |
| 233375_at | EFCAB2 | 0.48 | 0.023496 |
| 227379_at | MBOAT1 | 0.49 | 0.047911 |
| 225060_at | LRP11 | 0.49 | 0.046043 |
| 226354_at | LACTB | 0.49 | 0.030499 |
| 225466_at | PATL1 | 0.49 | 0.034083 |
| 218007_s_at | RPS27L | 0.50 | 0.031622 |
| 226310_at | RICTOR | 0.50 | 0.047972 |

FDR: false discovery rate
